# Abstracts from the 2026 joint meeting between MedStar Georgetown University Hospital's Functional Diabetic Limb Salvage: A Team Approach in collaboration with the Wound Healing Foundation, 8‐11^th^ April 2026, Washington, DC, USA

**DOI:** 10.1111/iwj.71008

**Published:** 2026-09-03

**Authors:** Laura K. S. Parnell, John Steinberg

**Affiliations:** ^1^ Wound Healing Foundation Barrington RI US; ^2^ Precision Consulting Missouri City TX US; ^3^ MedStar Georgetown University Hospital Washington DC US

MedStar Georgetown University Hospital's Functional Diabetic Limb Salvage and the Wound Healing Foundation, both nonprofit organizations, recently held their 2026 meeting on the diabetic foot and healing chronic wounds in Washington D.C., USA. These groups chose the International Wound Journal to feature their submitted abstracts to highlight the excellent research ongoing in this important clinical area. If you wish to contact any of the authors to discuss their research, please contact us and we can connect you.

The top ten abstract authors, denoted below in Abstracts 1‐10, were given podium presentations during the meeting in honor of Greg Schultz, PhD. At the time of his passing, Dr. Schultz was the Award Chair and a Board of Director of the Wound Healing Foundation and a Course Director for the joint meeting. Dr. Schultz was a consistent champion for quality clinical and bench science focused on improving wound healing. Presenting the best science in Dr Schultz's honor at the conference seemed like a natural extension to his legacy. Monitor https://www.woundhealingfoundation.org/g‐schultz‐in‐memoriam‐2/ for updated information on a future additional program honoring Professor Shultz in accordance with his family's wishes.

Abstracts selected to be included in conference proceedings as podium presentations are denoted below with the ^ɫ^ symbol.

Editorial Note: We recognize the valued contribution of Professor Greg Schultz and his connection with the IWJ. We are pleased to be able to continue honoring his legacy in wound healing.

## 1 Top 10 abstracts recognized in honor of Gregory Schultz, PhD

2

### 2.1 Correlation Between Positive MRI Findings and Bone Biopsy Results: A Retrospective Review

#### 2.1.1 Romeo Vences‐Leonard, DPM; Jacqueline Donovan, DPM; Danielle Butto, DPM; Diaeddine Madi, DPM; Jacob Wielgomas, MA

**Background:** Magnetic resonance imaging (MRI) is widely regarded as the most sensitive modality for diagnosing pedal osteomyelitis; however, false positives can occur due to reactive marrow changes and noninfectious etiologies. Bone biopsy with histopathology remains the diagnostic gold standard. This study aimed to evaluate the correlation between MRI findings positive for osteomyelitis and confirmatory bone biopsy results.

**Methods:** A retrospective review of 57 patients (July 2022–June 2025) who underwent foot MRI suggestive of osteomyelitis followed by bone biopsy was performed. Demographics, biopsy site, and imaging data were collected. MRI findings were compared with biopsy results to assess diagnostic accuracy. Sensitivity, specificity, PPV, and NPV were calculated, and significance among MRI, bone culture, and histology was tested using Cochran's Q and McNemar tests.

**Results:** Among 42 patients with MRI findings positive for pedal osteomyelitis, 22 (52.4%) were biopsy‐confirmed, 29 (69.1%) showed histologic infection, and 12 (28.6%) had no histologic evidence. MRI–biopsy concordance was 62.9%. MRI showed 100% sensitivity, 0% specificity, PPV 62.9% (vs. culture) and 70.3% (vs. histology), and NPV 0%. Cochran's Q test showed significant diagnostic differences; McNemar's test confirmed significance between MRI and biopsy (p=0.004) and MRI and histology (p=0.0012).

**Conclusions:** MRI frequently suggests pedal osteomyelitis, but a substantial proportion of positive cases are not confirmed by bone biopsy. This discrepancy underscores the necessity of histopathologic confirmation to guide surgical decision‐making and long‐term antibiotic therapy. Exclusive reliance on MRI may result in overdiagnosis and overtreatment.

3

### 3.1 Primary Targeted Muscle Reinnervation Confers Equivalent Gait Mechanics Compared With Below‐Knee Amputation Alone

#### 3.1.1 Luke Llaurado, MD; Jei Shih, MD; Kishan Shah, MD; Pilar Wisor, MD; Maryam Ganderi, MD

**Background:** Below‐knee amputation significantly affects an individual's quality of life, and restoring a functional gait in BKA patients is a primary rehabilitation goal. Targeted muscle reinnervation is a microsurgical technique that redirects transected sensory nerves into motor nerves to treat neuropathic pain, resulting in a less painful BKA stump for prosthetic use. Wearable sensors were used to quantitatively compare gait parameters between patients who had undergone BKA with and without TMR.

**Methods:** From June 2021 to June 2025, adult BKA patients meeting ambulation, wound‐free, and 3‐month postoperative criteria underwent gait testing. Using wearable sensors, patients completed a 120‐second walk and 30‐second Romberg test. Gait speed, mid‐swing elevation, step duration, sway, cadence, single‐ and double‐limb support, and stride length were compared between BKA and BKA+TMR groups. Demographics and LEFS scores were collected and compared between groups.

**Results:** Thirty‐seven BKA patients completed gait testing, including 22 with TMR and 15 without. Groups were similar in age, BMI, and comorbidity burden, though diabetes was more common with TMR. TMR group had a more recent surgery on average (1.9 ± 1.5 vs 8.7 ± 5.7 years; p < 0.001). No differences were found in gait, sway, or LEFS scores. TMR was not independently associated with outcomes, while greater time since surgery improved gait speed, stride length, limb support, and function.

**Conclusions:** In this pilot analysis of nontraumatic below‐knee amputees, primary TMR did not result in statistically significant differences in gait parameters or functional scores when compared to BKA alone. These findings suggest that, within this sample, TMR does not independently affect gait mechanics as measured by wearable sensors. Further studies with larger cohorts and longer follow‐up are needed to clarify the potential functional impact of primary TMR in this population.

4

### 4.1 Activating Progenitor Cells and Dermal Avn Microcirculation Through a Gargorized Wound Environment Reverses Ischemia and Necrosis, Enabling Amputation Prevention and Functional Limb Restoration.

#### 4.1.1 Bhuvneshwar Garg, MD, PhD, MS

**Background:** Despite modern strategies, diabetic foot disease continues to drive huge global morbidity, mortality and amputations. Care remains dominated by peripheral arterial disease paradigms, while dermal arteriovenous plexuses, capable of sustaining distal perfusion, are largely ignored. Emerging clinical evidence suggests that many “ischemic” wounds represent reversible biological ischemia due to functional suppression of dermal AVN microcirculation rather than irreversible vessel loss.

**Methods:** 61 high‐risk diabetic patients requiring immediate toe/foot amputation received Gargorization protocol that reawakens dermal AVN architecture and triggers Progen (G) cells. Routine antibiotics and oral hypoglycemics were withdrawn with physiologic insulin support as per need. Pus culture and D‐dimer served as baseline and recovery markers. Bioactive Super‐Curcumin dressings were used to remodel the wound microenvironment, restore endothelial signalling, and support mitochondrial recovery.

**Results:** Wound viability with necrosis reversal appeared within 72 hours, indicating wound remodelling and reawakening of dermal AVN microcirculation with sensory recovery in an average 10 days time. By the time pus culture reports available, wounds were clean in majority of patients. More than 90% limbs recovered as full functional weight‐bearing ones in an average 10 weeks time without antibiotics, major grafting, or angioplasty. Visual and whatsapp records secured for follow ups and documentations.

**Conclusions:** Reconditioning the wound into a Gargorised regenerative milieu can reactivate endogenous progenitor cells, restore AVN microcirculation with rapid reversal of ischemia and necrosis. Delivered through a scalable, antibiotic‐sparing “Healing‐at‐Home” model, it promotes functional limb restoration and amputation prevention. Signalling a paradigm shift from vessel to microcirculation‐centric limb salvage, it is easy to deploy, cost‐efficient, and employment generator too in low‐resource settings.

5

### 5.1 Effectiveness of Human Cellular Umbilical Tissue Compared to Xenografts in Managing Diabetic Lower Extremity Ulcers in Hospitalized Patients

#### 5.1.1 Chien Chang Chen, PhD; Jaideep Banerjee, PhD; Sarah Richards, DPM; Tim Styche

**Background:** Due to lack of sufficient statistical difference in clinical outcomes between broad categories of CAMPs, decision makers should consider head‐to‐head comparative studies and real‐world evidence between individual CAMPs to decide on which to use in practice. The objective of this study was to compare the clinical and economic outcomes of hospital care for DLEU patients treated with CAMPs

**Methods:** This real‐world analysis was conducted on patients with DLEU treated with CAMPs between January 2017 to March 2025 using data from the Premier PINC AI Healthcare Database. Treatment arms included a human cellular umbilical tissue (hcUT) and four acellular xenografts. IPTW‐adjusted generalized linear models were utilized to estimate healthcare‐related outcomes and hospital‐related costs.

**Results:** 2,350 DLEUs patients with hcUT and 8,786 DLEUs patients with xenografts were identified. Patients treated with the hcUT had 9% higher odds of being discharged to home (OR=1.09, p=0.0023) and a 6% shorter length of stay (Ratio=0.94, p<0.0001). Additionally, patients receiving hcUT had lower odds of having an all‐cause inpatient or outpatient visit (OR=0.86, p<0.0001), a dehiscence‐related visit (OR=0.81, p=0.0004), a visit with a debridement procedure (OR=0.83, p<0.0001), a minor amputation (OR=0.88, p=0.0001), or a major amputation (OR=0.82, p=<0.0001) within 6 months after the index admission. Patients with hcUT had an 11% lower total cost (Ratio=0.89; p<0.0001) during the index admission.

**Conclusions:** This large dataset demonstrates that patients receiving hcUT had improved clinical outcomes and may be more cost‐effective in managing DLEU compared to some xenografts.

6

### 6.1 Gait Asymmetry as an Independent Predictor of Ulceration in Patients with Below‐Knee Amputations

#### 6.1.1 Stella Kim, BS; Jie Jung Shih, MS; Maryam Ghaderi, MS; Hannah Soltani, BA; Karen Kim Evans, MD; Richard Youn, MD; John Steinberg, DPM; Jayson Atves, DPM; Christopher Attinger, MD

**Background:** Existing literature details gait instability in patients with lower limb amputations, demonstrating slower walking velocity, increased variability of angular velocity patterns, and altered regularity. Gait speed was considered a significant predictor for plantar loading and pressure that increases the risk of ulcerations. Yet, less attention has been paid to the relationship between gait symmetry and ulcerations, especially in patients with lower limb amputations.

**Methods:** Symmetry index was calculated from gait parameters with formula proposed by Herzog et al., computed as twice the difference between right and left limbs divided by the sum of the parameters. Patients with >20% difference on any of the following parameters gait speed, step duration, cadence, single limb support, double limb support, or stride were classified as asymmetry group, patients without any were classified as symmetry group.

**Results:** A total of 39 patients were included, with 15 (38.5%) in the symmetry group and 24 (61.5%) in the asymmetry group. Baseline characteristics did not differ between groups in age, BMI, smoking status, diabetes, peripheral neuropathy, PVD, CKD, or Charlson Comorbidity Index (all p>0.05). Ulceration was significantly more prevalent in the asymmetry group (73.3% vs 95.8%, p = 0.042). On multivariate analysis, gait asymmetry (p=0.014) and PVD (p=0.017) were independently associated with ulceration.

**Conclusions:** Gait asymmetry in below‐knee amputees is independently associated with increased ulceration risk. Dynamic analysis of gait is important feedback for physical therapy and prosthetists. We suggest a multidisciplinary team clinical evaluation that combines gait analysis with prosthesis fitting to prevent ulcerations.

7

### 7.1 HbA1c Does Not Predict Lower Extremity Amputation Risk in Patients with End Stage Renal Disease on Hemodialysis

#### 7.1.1 Nichole Andrade; Hannah Soltani, BA; Kenneth Fan, MD; Richard Youn, MD; Christopher Attinger, MD; Karen Kim Evans, MD

**Background:** Patients with end tage renal disease on hemodialysis face high lower extremity amputation risk. Risk stratification commonly relies on an HbA1c threshold of 7%, yet anemia, accelerated red blood cell turnover, and uremia may mask true glycemic burden. Despite KDOQI guidance acknowledging HbA1c underestimation in ESRD, consensus targets remain unclear. We evaluated whether HbA1c reliably predicts limb loss risk in this population.

**Methods:** We performed a retrospective multicenter study of 541 patients with ESRD on hemodialysis from 2012 to 2022 across four tertiary hospitals. The association between HbA1c and lower extremity amputation was evaluated modeling HbA1c continuously and categorically. Multivariable logistic regression adjusted for demographics, BMI, diabetes, and smoking. Predicted probabilities were estimated with marginal effects analysis.Discrimination was assessed with ROC analysis versus diabetes diagnosis alone.

**Results:** HbA1c, tested as a continuous variable (OR 1.09 per 1% increase, p=0.13), at the standard 7% cutoff (OR 1.57, p=0.16), and across half‐point increments, showed no significant association with LEA in either odds ratios or predicted probabilities. HbA1c added no discriminatory power beyond diabetes diagnosis, with ROC performance nearly identical across continuous HbA1c (AUC 0.733), categorical HbA1c (0.727), and diabetes status alone (0.727).

**Conclusions:** In patients with ESRD on hemodialysis, HbA1c fails to stratify limb loss risk and offers no predictive advantage beyond a diabetes diagnosis. Reliance on HbA1c thresholds may underestimate true risk and delay timely vascular or reconstructive intervention. Development of ESRD‐specific glycemic and ischemic risk models is critical to improve limb salvage outcomes.

8

### 8.1 Predictors Of Ambulatory Recovery Following Nontraumatic Lower Extremity Reconstruction: The Role Of Comorbidity, Reconstruction, And Discharge Planning

#### 8.1.1 Hannah Soltani, BA; Christopher Ply, BS; Rachel Rohrich, BS; Richard Youn, MD; Karen Kim Evans, MD; Christopher Attinger, MD

**Background:** Ambulation is a key marker of success in functional lower extremity (LE) reconstruction and limb salvage. While predictors of ambulation are well described for traumatic LE injury, they remain poorly defined for chronic, nontraumatic wounds. This study identifies factors associated with postoperative ambulation in a large, medically complex cohort.

**Methods:** We retrospectively reviewed 745 patients who underwent LE reconstruction with local flaps, free tissue transfer (FTT), or below‐knee amputation (BKA) for nontraumatic indications between 2011 and 2023. Patients were stratified by ambulatory status at final follow‐up. Median follow‐up was 25.6 months [IQR: 43.5]. Clinical, reconstructive, and discharge‐related variables were analyzed using multivariable logistic regression to identify independent predictors of ambulation.

**Results:** At final follow‐up, 511 (68.6%) patients were ambulatory. NA status was most common in BKAs (62.0% vs. 30.1% in FTT, p<0.001). Preoperative ambulation (OR: 3.8, p<0.001) and FTT (OR: 1.9, p=0.032) were independent predictors of ambulation, while smoking (OR: 0.5, p=0.005), coronary artery disease (OR: 0.6, p=0.045), and discharge to skilled nursing or subacute rehabilitation rather than acute rehabilitation (OR 0.2 and 0.5, respectively) were associated with reduced odds of ambulation.

**Conclusions:** Baseline function, reconstructive approach, and discharge disposition strongly predict ambulation in nontraumatic LE reconstruction, supporting tailored multidisciplinary care.

9

### 9.1 Co‐surgeon Approach Allows for Expanded Indications for Lower Extremity Free Tissue Transfer

#### 9.1.1 Hannah Soltani, BA; Christopher M. Ply, BS; Rachel N. Rohrich, BS; Jessica R. Ong, MD PhD; Leila I. Jones, BA; Richard C. Youn, MD; Christopher E. Attinger, MD; Karen K.Evans, MD

**Background:** Lower extremity (LE) free tissue transfer (FTT) is often performed in highly comorbid patients with chronic wounds and poor vascular supply, which may limit candidacy for reconstruction. The co‐surgeon approach, involving two surgeons simultaneously performing a single procedure, has been shown to improve operative efficiency and patient outcomes in breast reconstruction and spine surgery, yet its role in LE FTT has not yet been explored.

**Methods:** A retrospective review of patients undergoing LE FTT from July 2011‐June 2025 was conducted. Patients were stratified into two groups: single surgeon vs co‐surgeon approach (adopted in August 2022). A total of 423 underwent LE FTT, of which 141 (33.3%) utilized the co‐surgeon approach. Comorbidities (including chronic kidney disease [CKD] and peripheral vascular disease [PVD]), wound characteristics, operative details, and postoperative outcomes were compared.

**Results:** Upon adoption of the co‐surgeon approach, annual surgical volume increased by 35%. Patients in the co‐surgeon group were older (median 61.5 vs 57 years, p=0.006), had higher BMIs (median 30.6 vs 28.5 kg/m2, p=0.023), higher rates of CKD (19.1% vs 10.3%, p=0.010) and PVD (64.5% vs 46.4%, p=0.018). The co‐surgeon group utilized more complex flap types (p<0.001), including omental flaps (2.8% vs 0%), yet had comparable length of stay (median 26 [IQR: 16] days) and flap success (95.7%).

**Conclusions:** The co‐surgeon approach confers a novel benefit in LE FTT by enabling safe expansion of surgical candidacy and operative volume to include more medically complex patients requiring complex flaps, without increasing operative duration or major complications.

10

### 10.1 Continuous Tissue Oximetry Is Associated with Improved Free Flap Salvage Rates After Operative Re‐Exploration in Chronic Lower Extremity Reconstruction

#### 10.1.1 Christopher Ply, BS; Sasha Lasky, MD; Hannah Soltani, BA; Rachel Rohrich, BS; Meghan Currin, BA; Cameron Akbari, MD; Richard Youn, MD; Christopher Attinger, MD; Karen Kim Evans, MD

**Background:** Acute compromise of lower extremity (LE) free tissue transfer (FTT) remains a devastating complication, with lower reported salvage rates compared to other reconstructive sites. Data examining factors associated with successful salvage following operative re‐exploration in patients undergoing FTT for chronic LE wounds are limited. This study aimed to identify factors associated with successful flap salvage after urgent return to the operating room for lower extremity free flap compromise.

**Methods:** A retrospective review of all LE FTTs performed for chronic wounds at a single tertiary referral center from July 2011 to November 2025 was conducted. Free flaps with an emergent return to the operating room for acute flap compromise with intent to salvage were included. Flaps were stratified into successful salvage and salvage failure. Demographics, comorbidities, vascular characteristics, flap details, and outcomes were compared.

**Results:** Among 409 LE FTTs, 25 (6.1%) required operative re‐exploration, of which 19 (76.0%) were successfully salvaged. Continuous tissue oximetry monitoring with ViOptix was more frequently used in salvaged flaps (73.7% vs. 16.7%, p=0.013) and was associated with shorter time to operative re‐exploration. ViOptix monitoring was independently associated with increased odds of successful salvage of cutaneous free flaps (OR 16.4, 95% CI 1.4–199.6; p=0.028).

**Conclusions:** This is the first report analyzing ViOptix use on flap salvage rates in lower extremity free flap patients. Early detection of flap compromise facilitated by tissue oximetry may enable more timely re‐exploration and improved salvage in high‐risk LE reconstruction, preventing a second free flap surgery or limb amputation. Additionally, more flaps monitored with ViOptix were admitted to the surgical floor rather than intensive care unit postoperatively.

11

### 11.1 Feed the Flap, Save the Limb, Save the Life: The Impact of Preoperative Malnutrition on Lower Extremity Free Tissue Transfer for Atraumatic Limb Salvage

#### 11.1.1 ^Δ^Finn Tobias, BA; Hannah Soltani BS; Rachel N. Rohrich, BS; Christopher M. Ply, BS; Sami Ferdousian, MS; Ryan P. Lin, MD; Meghan E. Currin, BA; Cameron M. Akbari, MD; Richard C. Youn, MD; Christopher E Attinger, MD; Karen K. Evans, MD

**Background:** Nutritional status plays is well known to modulate wound healing. Patients requiring lower extremity (LE) free tissue transfer (FTT) often experience malnutrition. This study evaluates the impact of perioperative malnutrition on flap complications and long‐term limb salvage among patients undergoing microsurgical FTT for atraumatic limb salvage.

**Methods:** A retrospective review of all LE FTT performed for chronic wounds from February 2011 to August 2024 was conducted. Patients with available preoperative nutritional labs (albumin and prealbumin) within 7 days of FTT were included. Malnutrition was defined using established cutoffs: hypoalbuminemia as <3.5 mg/dL and low prealbumin as <16 mg/dL.

**Results:** Among 253 patients, 69.2% had hypoalbuminemia and 34.8% low prealbumin. Low prealbumin (<16 mg/dL) was linked to more flap complications (p=0.015), including partial necrosis (p=0.010) and infection (p=0.047), without affecting flap success. Hypoalbuminemia (<3.5 mg/dL) was associated with lower limb salvage rates (p=0.017) and higher mortality (p=0.012), independently predicting amputation (OR 3.25, p=0.031), mortality (OR 12.7, p=0.023), and flap complications (OR 1.9, p=0.046).

**Conclusions:** Perioperative nutritional status holds significant prognostic value for adverse outcomes following LE FTT. Low prealbumin, which may reflect acute malnutrition, appears to contribute to flap‐related complications, whereas hypoalbuminemia, which may reflect chronic malnutrition, correlates with long‐term limb loss and mortality. Optimizing nutritional status preoperatively and longitudinal maintenance postoperatively may improve limb salvage and overall outcomes in this high‐risk population.

12

### 12.1 Prevalence and Screening Gaps for Vitamin D Deficiency in the Limb Salvage Population

#### 12.1.1 Hannah Soltani, BA; ^Δ^Cameron Darnell, BS; Ryan Bender, MD; Christopher Ply, BS; Jayson Atves, DPM; John S. Steinberg, MD; Christopher E. Attinger, MD; Richard C. Youn, MD; Karen K. Evans, MD

**Background:** Vitamin D deficiency plays a critical role in immune modulation, inflammation control, and tissue repair. Impaired vitamin D status may contribute to delayed healing, infection risk, and poor reconstructive outcomes. The prevalence and screening practices of vitamin D deficiency in this chronic lower extremity wound population remain poorly characterized.

**Methods:** We performed a retrospective review of all patients undergoing lower extremity reconstruction or amputation at our institution between July 2011 and June 2025. Preoperative vitamin D levels, when available, were recorded. Cohorts included free tissue transfer (FTT), local flaps (LF), and below‐knee amputations (BKA). Deficiency was defined per institutional laboratory reference ranges. Screening frequency and prevalence of deficiency were analyzed across groups.

**Results:** A total of 951 patients were screened at a median of 6.1 months prior to surgery, of which 44.9% received FTTs, 16.3% received LFs, and 38.8% received BKAs. Among patients receiving FTTs (n=427), 16 patients (3.7%) were screened and 6 (38%) were deficient. Of 155 patients receiving LFs, 18 (11.6%) were tested, with 7 (39%) deficient. In 369 patients undergoing BKA, 54 patients (14.6%) had levels recorded, and 31 (57%) were deficient. Overall, deficiency affected 44 of 88 screened patients (50%).

**Conclusions:** Vitamin D deficiency is highly prevalent in patients with chronic lower extremity wounds but is infrequently assessed preoperatively. Given its role in wound healing and the ease of supplementation, routine screening represents a simple opportunity to optimize outcomes. Based on these findings, our institution has now implemented standardized vitamin D screening for all limb salvage and amputation patients.

## 13 ABSTRACT POSTERS

## 14 Poster numbers are identified in the order they were submitted

15

### 15.1 How we are distributed as a profession: Analysis of the National Downloadable File and US census data

#### 15.1.1 Jessica Carrillo, DPM; Daniel Ruggiero, DPM; Daria Zucchi, DPM; Andrew Meyr, DPM

**Background:** The objective of this investigation was to evaluate the distribution of contemporary DPMs based on socioeconomic and disease variables.

**Methods:** The National Downloadable File provides demographics of all physicians with an active NPI. Data related to those physicians with a degree of “DPM” was extracted. The database was further refined to analyze the number of DPM billing addresses per US state and zip code. Mean and median income per state was accessed through 2023 US census data, and the number of residents per state diagnosed with diabetes mellitus was accessed through the American Diabetes Association.

**Results:** A statistically significant positive correlation was observed between the number of DPM billing address per zip code with both median (0.084, p<0.001) and mean (0.094, p<0.001) income. We further observed a mean±standard deviation (range) of 2582.1±1593.8 (824‐10148) ratio of state DPMs to state residents with diabetes mellitus. The 5 areas with the highest disparate ratios were: Puerto Rico (10148:1), Mississippi (8056:1), Arkansas (4754:1), Alabama (4652:1), South Carolina (3994:1).

**Conclusions:** Results of this investigation provide unique information of the distribution of contemporary DPMs in the US based on socioeconomic and disease variables. Several likely underrepresented areas were identified and might represent areas of growth for the podiatric profession.

16

### 16.1 Access to Podiatric Care in Pennsylvania

#### 16.1.1 Sahil Kapadia; Posi Asasolu; Daniel Ruggiero, DPM; Andrew Meyr, DPM

**Background:** The objective of this investigation was to examine the prevalence of DPMs in Pennsylvania and assess the availability of podiatric care to at‐risk patients in the state based on zip code.

**Methods:** The National Downloadable File provides demographics of all physicians with an active NPI. The database was refined to analyze the number of DPM billing addresses per PA zip code. Mean and median income per state was accessed through 2023 US census data, and the number of residents per state diagnosed with diabetes mellitus was accessed through the American Diabetes Association.

**Results:** We identified 372 zip codes in PA with at least one DPM billing address with a mean±standard deviation (range) of 4.2±5.6 (1‐72) billing addresses per zip code. We identified 912 DPMs with an active NPI in July of 2025 with a primary billing address in Pennsylvania. We observed a positive Pearson correlation coefficient (0.368; p<0.001) between the number of DPM billings addresses in a zip code and the population of that zip code.

**Conclusions:** We found that Pennsylvania was a relatively "old" state when considering the national demographics of podiatric physicians, as well as positive correlations between DPM billing addresses with zip code population, mean income and median income. Relatively positive diabetic patient access to podiatric care was observed relative to national averages.

17

### 17.1 A Dual‐Modality Approach for DFU Repair: NPWT Plus Fespixon Cream Improves Healing Dynamics DFU

#### 17.1.1 Tianbo Li, MD; Lei Gao, MD

**Background:** Diabetic foot ulcers (DFUs) remain challenging to heal and often require adjunctive local therapies to optimize wound bed preparation.

**Methods:** This retrospective study included 80 DFU patients treated at Beijing Shijitan Hospital, Capital Medical University (November 2024–February 2025). Patients receiving NPWT alone were assigned to the control group (n=37), and those receiving NPWT + Fespixon Cream were assigned to the combination group (n=43). Baseline characteristics were comparable between groups (P>0.05). Outcomes assessed included ulcer healing rate, granulation tissue coverage, total and serious adverse events (AEs), and quality‐of‐life (QoL) scores.

**Results:** Baseline ulcer healing rate did not differ between groups (P>0.05). From week 2 onward, healing rates increased in both groups, with the combination group showing significantly higher healing rates than the control (P<0.05). Granulation tissue coverage was similar at baseline (P>0.05) and improved progressively at weeks 1–4 in both groups; the combination group consistently demonstrated higher granulation coverage at each time point (P<0.05). The total AE rate was 24.32% (control) vs. 16.28% (combination), and serious AE rates were 5.41% vs. 2.33%, with no significant differences (P>0.05). QoL scores improved significantly in both groups by week 4, with greater improvement in the combination group (P<0.05).

**Conclusions:** Fespixon Cream combined with NPWT was associated with faster DFU healing and enhanced early granulation formation, and better QoL improvement, without increasing adverse event risk, compared with NPWT alone.

18

### 18.1 A Systematic Review of Systemic Treatment Options for Necrobiosis Lipoidica

#### 18.1.1 Sonam Mistry; Michael Payette

**Background:** Necrobiosis lipoidica (NL) is a rare granulomatous inflammatory condition often associated with diabetes and disproportionately affects the lower extremities. Lesions typically present as indurated, erythematous plaques or papules that may progress to atrophy and ulceration. Although topical, photodynamic, and ultraviolet therapy may be used, many cases are refractory, and evidence supporting systemic treatment options remains limited. This systematic review evaluates systemic therapies for NL.

**Methods:** A MeSH‐based systematic search of PubMed and Cochrane databases was conducted using the terms “necrobiosis lipoidica” and “therapeutics.” Primary reports published between 2005 and 2025 describing systemic biologic or oral therapies were included. Extracted data included the therapeutic agent, degree of lesion improvement, time to response, and reported adverse events.

**Results:** Among 81 screened articles, 28 case reports met inclusion criteria. 43/49 patients demonstrated clinical improvement or resolution with systemic therapy. Reported agents included pentoxifylline (2), Janus kinase inhibitors (4), secukinumab (1), ustekinumab (3), doxycycline (1), tumor necrosis factor–a inhibitors (7), antimalarials (2), fumaric acid esters (1), intravenous immunoglobulin (1), dipyridamole (1), combined immunosuppressives (1), autografting (1), and pancreatic‐kidney transplantation (3). Reported adverse events were generally mild and included gastrointestinal symptoms, fatigue, dizziness, rash, infections, and localized edema.

**Conclusions:** Systemic therapies may play a critical role for promoting lesion stabilization and ulcer healing in NL, potentially reducing the risk of limb‐threatening complications especially in diabetic patients. Early identification and initiation of systemic therapy may decrease pain, resolve ulcerations, and avoid chronic disfigurement. Prospective studies and clinical trials are needed to guide evidence‐based treatment strategies for NL.

19

### 19.1 Evaluating Endovascular Approach vs. Open Revascularization Approach for Patients with Chronic Limb‐Threatening Ischemia needing Diabetic Limb Salvage

#### 19.1.1 Apurwa Shah; Sahil Kapadia; Celine Mbah; Taylor Champ; Mahekkumar Bhut

**Background:** Chronic limb threatening ischemia (CLTI) is the leading cause of major foot amputation in patients with diabetes mellitus (DM). This review evaluates and compares the limb salvage related outcomes after endovascular versus open surgical revascularization in patients with DM and CLTI by focusing on the major amputation, amputation‐free survival, wound healing, and the need for re‐intervention.

**Methods:** Studies within the last 10 years were evaluated to assess major amputation, amputation‐free survival, wound healing, and reintervention rates. The study focused on adult patients with DM and CLTI mostly presenting with ischemic tissue loss, including diabetic foot ulcers or gangrene. Studies with non‐diabetic populations and without limb salvage intent were excluded. Initial search yielded 14 studies, title filtration yielded 10 studies and abstract filtration yielded 8 studies.

**Results:** Across the eight studies, major amputation rates ranged from approximately 10‐25% at 1 year, regardless of revascularization technique. Amputation‐free survival improved with active revascularization and ranged from 65‐85%, largely influenced by ischemia severity, infection, and soft tissue pathology. Wound healing outcomes were variable but generally ranged from 65‐85% at 12 months. Reintervention was more common after endovascular revascularization (40%) compared with open bypass (30%).

**Conclusions:** Both endovascular and open surgical revascularization can achieve limb salvage in diabetic patients with CLTI, with neither approach showing clear superiority. Major amputation and amputation‐free survival are dependent on a patient's ischemia severity, wound, infection and timing of the revascularization rather than the technique. Endovascular therapy is associated with higher rates of reintervention, supporting an individualized, foot‐focused, multidisciplinary approach to limb salvage.

20

### 20.1 Home‐Based Exercise to Improve Functional Outcomes in Veterans With A Recently Healed Diabetic Foot Ulcer

#### 20.1.1 Mary‐Claire Roghmann, MD; Odessa Addison, DPT; Steve Prior, PhD; Kiana Trent, DPM; Brock Beamer, MD; Elizabeth Dennis, PhD, RD; Olivia Porcello; Bailey Rammling, MS; Gwen Robinson, MPH; Alison Lydecker, MPH; John Sorkin, MD, PhD; Gloria Izore, DNP

**Background:** A common complication of diabetes is diabetic foot ulcers (DFU) for which a mainstay of treatment is off‐loading and thus prolonged immobility. Those with healed DFU often fail to regain pre‐ulcer mobility, threatening functional independence in older adults. There is limited research on exercise during ulcer remission. Therefore, this pilot study assessed the feasibility and acceptability of a home‐based exercise regimen aimed at safely increasing mobility and function.

**Methods:** Veterans aged 50+ with healed plantar DFU were eligible: 26 enrolled; 20 were randomized to a 12‐week home‐based exercise regimen or standard of care (3:1 ratio) with baseline tests for gait speed, physical performance, and community mobility. The intervention group participated in virtual exercise classes twice a week led by the study team and home cycling three times a week. The control group received usual care. Outcome measures include feasibility, acceptability, and changes in gait speed, physical activity levels, and strength.

**Results:** At baseline, the average gait speed was 0.95 ± 0.27 (meters/second). A healthy gait speed for older adults is 1.2. Additionally, the average Modified Physical Performance Test total score was 30 ± 5 with a normal score greater than 32. The trial is on‐going with no exercise‐related adverse events.

**Conclusions:** We were successful in meeting enrollment goals. Baseline testing demonstrates low physical performance and suggests a high prevalence of frailty. If feasible and acceptable, the exercise intervention will be tested in a future multi‐site randomized clinical trial to assess its impact on mobility, cardiovascular health, and ulcer recurrence.

21

### 21.1 Cost Effectiveness Analysis Comparing Collagen Based Dressings and Skin Substitute Grafts in Treatment of Non‐Pressure Ulcerations

#### 21.1.1 Helena Romeo; James McGuire, DPM

**Background:** Skin substitutes (SS) have been used to enhance wound healing. Studies evaluating them have used a standard of care (SOC) of saline dressings. In this poster we attempt to compare the healing rates of these tissues to a “new SOC” (nSOC) using commonly utilized collagen dressings. The recent high profile SS abuse cases raise concerns about the ROI of using these products. This study compares healing outcomes and cost‐effectiveness of commonly used SS grafts using the nSOC.

**Methods:** Six of the most popular SS grafts were evaluated using healing rates at 12 weeks and estimated cost per 1cm^2^. Incremental Cost‐Effectiveness Ratios (ICERs) were then calculated to assess differences in cost relative to healing effectiveness compared to the nSOC as defined above. ICERs measure the additional cost per percent of wounds healed gained by using one treatment over another. Knowing a treatment's cost‐effectiveness helps physicians decide if a product's higher price is justified.

**Results:** Five of the six products demonstrated positive ICERs, indicating higher healing rates at increased cost, while Apligraf was associated with a negative ICER, reflecting lower cost and lower effectiveness. GraftJacket demonstrated the greatest improvement in healing rates relative to SOC. Substantial variability was observed across products, suggesting that higher cost does not consistently correlate with improved outcomes.

**Conclusions:** Overall, skin substitute products may improve wound healing compared to even the nSOC, but their cost‐effectiveness varies significantly by product. These findings highlight the importance of individualized clinical decision making that balances clinical benefit, financial considerations, and insurance coverage. Further research incorporating wound etiology, patient demographics, and long‐term outcomes is warranted to establish evidence‐based guidelines for optimal SS graft utilization.

22

### 22.1 Comparison of Healing and Adverse Events Following Dermal Regeneration Template Reconstruction in Acute and Chronic Lower Extremity Wounds

#### 22.1.1 Stephanie Mueller; LaYow Yu; Katherine Goundry; Devin Clegg; Dennis Orgill

**Background:** Dermal Regeneration Templates (DRTs) are skin substitutes consisting of bovine collagen‐glycosaminoglycan matrix and an outer silicone layer. While DRTs have proven efficacy in acute wounds and diabetic foot ulcers (DFUs), comparative outcomes between chronic and acute lower extremity wounds remain poorly defined. This study compares healing outcomes and adverse events (AEs) following DRT placement across lower extremity wound etiologies.

**Methods:** A retrospective cohort study was performed including patients from two academic institutions who underwent Integra® DRT placement to the distal lower extremity wounds (2015‐2024). Outcomes included one‐year wound healing and AEs, defined as DRT removal, unplanned flap reconstruction, or amputation. Analysis included Cox proportion hazards modeling and chi‐square or Fisher's exact testing.

**Results:** 195 patients were included. Chronic wounds (n=53) included arterial ulcers, DFUs, venous leg ulcers, and chronic wounds of unclear etiology. Acute wounds (n=142) included oncologic resection, trauma, donor site coverage, and burns. One‐year AE rates were 58% in chronic wounds and 26% in acute wounds; one‐year healing rates were 34% and 77%, respectively. Chronic wounds had a lower likelihood of healing (HR 0.22, 95% CI 0.08–0.58, P=0.002) and increased odds of AEs (OR 7.04, P=0.001).

**Conclusions:** Outcomes following DRT placement vary by wound chronicity. Chronic lower extremity wounds are associated with delayed healing and increased adverse events at one year compared with acute wounds.

23

### 23.1 Optimizing Antibiotic Therapy Following Toe and Transmetatarsal Amputation in Diabetic Foot Osteomyelitis

#### 23.1.1 Netanya Flores, DPM, MS

**Background:** Diabetic foot osteomyelitis (DFO) is a limb‐threatening infection often requiring minor amputation. The 2023 IWGDF/IDSA guidelines advocate margin‐directed therapy: shorter courses after complete infected bone resection, up to 3 weeks for positive proximal margins, and prolonged 6‐week therapy only when bone remains unresected. Emerging randomized and comparative evidence suggests shorter, oral‐based regimens can achieve comparable infection control and limb preservation.

**Methods:** We conducted a systematic review of PubMed, Embase, Web of Science, and Google Scholar (2014–2024) identifying randomized trials, cohort studies, and systematic reviews on antibiotic duration after partial foot amputation for DFO. Search terms included diabetic foot osteomyelitis, minor amputation, bone margin status, and antibiotic duration. Guideline statements and landmark duration and route‐of‐therapy trials were included for contextual analysis.

**Results:** We conducted a systematic review of PubMed, Embase, Web of Science, and Google Scholar (2014–2024) identifying randomized trials, cohort studies, and systematic reviews on antibiotic duration after partial foot amputation for DFO. Search terms included diabetic foot osteomyelitis, minor amputation, bone margin status, and antibiotic duration. Guideline statements and landmark duration and route‐of‐therapy trials were included for contextual analysis.

**Conclusions:** Margin‐guided, shorter antibiotic courses after minor amputation for DFO provide effective infection control and limb preservation while minimizing toxicity. Implementing standardized, margin‐based protocols enhances limb salvage outcomes and aligns practice with antimicrobial stewardship principles.

24

### 24.1 An Initial Study: Among Patients Undergoing Lower Extremity Revascularization for Pad/Clti, do Outcomes Differ Based on how Soon a Podiatric Team Becomes Involved After the Index Vascular Intervention?

#### 24.1.1 Sahil Kapadia; Posi Asaolu; Chinkal Patel; Amarriah Valentine, MS; Janeen Elagha, DPM

**Background:** Patients with PAD/CLTI remain at high risk for nonhealing ulcers and amputation despite lower extremity revascularization. Multidisciplinary models integrating vascular and podiatric care improve limb salvage and are economically viable, but structures vary by institution and may influence quality of care.

**Methods:** This retrospective cohort study at a two‐hospital academic system evaluated adults undergoing infra inguinal revascularization for PAD/CLTI, grouping limbs by timing of podiatric involvement (same day, 0–7, 8–30, >30 days, or none within 6 months). The first vascular intervention defined the index event, and patients with prior major amputation of the same limb or initial podiatry consultation were excluded. Primary outcomes were major amputation and one year amputation‐free survival.

**Results:** In the initial chart review, same‐day podiatry involvement was associated with 100% amputation‐free survival, compared with approximately 50% when podiatry became involved within 7 days. Vascular procedures were most often diagnostic angiography or endovascular interventions. Wound healing outcomes after podiatric intervention varied but were favorable among compliant patients, and no repeat vascular procedures occurred within 12 months when podiatry was involved within the first week.

**Conclusions:** Early podiatric co‐management appears to support improved limb preservation and resource utilization. However, these findings arise from an established vascular–podiatric team, which may bias outcomes toward better coordination than in less integrated settings. Larger multicenter studies across diverse hospital systems with varying vascular–podiatric relationships are needed to validate these associations and define generalizable, evidence‐based targets for timely podiatric integration.

25

### 25.1 Post‐Angioplasty Diabetic Foot Attack: Recombinant Human Epidermal Growth Factor(rhEGF) Versus Conventional Therapy in a Pilot Comparative Case Series

#### 25.1.1 Rafael Cendejas, MD; Erika Celestino, RN, BSN; Diana Camarillo, RN

**Background:** Endovascular revascularization improves perfusion in ischemic diabetic foot attack (DFA); however, wound healing often remains prolonged despite technical success. Adjunctive therapies aimed at enhancing tissue repair may improve healing trajectories and limb salvage outcomes after angioplasty.

**Methods:** This pilot comparative case series included six male patients with diabetic foot attack who underwent successful endovascular angioplasty. Three patients received intralesional rhEGF and standard wound care, three received conventional therapy alone. Baseline wound severity was assessed using the WIfI and SEWSS. Tissue perfusion was evaluated using TcpO2 before/after angioplasty. Primary endpoint: time to complete epithelialization. Secondary: time to adequate granulation and TcpO2 changes.

**Results:** Patients with rhEGF presented greater baseline ischemic severity, with lower pre‐angioplasty TcpO2 values and higher WIfI and SEWSS stages. rhEGF group demonstrated earlier granulation(6–12 weeks) compared with the conventional group(20–32 weeks). Median time to complete epithelialization was comparable between groups despite increased baseline severity in the rhEGF cohort. Improvement in TcpO2 following angioplasty was greater increases in rhEGFgroup. No major amputation in both groups.

**Conclusions:** In this pilot comparative case series, adjunctive use of rhEGF after endovascular revascularization was associated with earlier granulation and favorable wound healing trajectories despite greater baseline ischemic severity. These exploratory findings support further evaluation in larger prospective studies.

26

### 26.1 Performance of Fetal Bovine Dermis in Diabetic Foot Ulcers: Insights From Real‐World Clinical Use

#### 26.1.1 Yifei Dai, MS; Jessica Evans, MD; Maria Leonard RN, BSN; Yi Arnold, PhD; Paula Searcy

**Background:** Diabetic foot ulcers (DFUs) are challenging chronic wounds to manage. Biologic matrices such as fetal bovine dermis (FBD) provide structural support that may promote tissue regeneration. Prior randomized data have shown higher 12‐week healing rates with FBD compared with standard care. Despite these results, real world evidence across diverse clinical environments remains limited. This study evaluates routine clinical outcomes for DFUs treated with FBD in the United States and Europe.

**Methods:** A retrospective chart review was conducted using a HIPAA compliant online platform. Clinicians submitted de identified cases received FBD between June 2022 and June 2025, with a maximum of 20 cases per contributor. Standardized data fields captured wound classification, time to closure, healing status, and safety events. The retrospective and anonymized nature of the project qualified it for exemption from consent requirements. Analysis of DFU cases consisted of descriptive statistics.

**Results:** A total of 123 DFU cases were included. Complete wound closure at 12 weeks occurred in 77.2% of cases (95/123). No device related intraoperative adverse events were reported. Infection was documented in 3.3% of cases, and DFU recurrence occurred in 6.5%, primarily within six months of initial healing.

**Conclusions:** This real‐world dataset demonstrates that FBD performs consistently in routine DFU management and shows outcomes comparable to those previously reported in randomized controlled trial The high closure rate and low complication profile support the utility of FBD in routine wound care. While retrospective design and variability in follow up represent limitations, findings suggest durable healing and potential reduction in downstream risks such as hospitalization and limb loss.

27

### 27.1 Real‐World Use of Fetal Bovine Dermis in Challenging Wound Presentations

#### 27.1.1 Yefie Dai, PhD; Maria Leonard, RN, BSN; Jessica Evans, MD; Yi Arnold, PhD; Paula Searcy

**Background:** Wounds featuring tunneling, undermining, or persistent drainage are difficult to manage due to irregular geometry and poor tissue contact, often leading to slow healing and increased infection risk. Biologic dermal matrices such as fetal bovine dermis (FBD) may improve integration in these environments. This study reviews real‐world outcomes of FBD in complex wounds across U.S. and EU practices.

**Methods:** A retrospective review was conducted using a HIPAA compliant platform. Clinicians entered de identified cases treated with FBD from June 2022 to June 2025. Each clinician could contribute up to 20 cases. Data included wound characteristics, healing at 12 weeks, adverse events, and recurrence. Only wounds with tunneling, undermining, or drainage were analyzed. The anonymized design met criteria for IRB exemption.

**Results:** Among 246 cases, 123 involved tunneling/undermining and 123 involved drainage. At 12 weeks, closure occurred in 85.4% of tunneled/undermined wounds and 81.3% of draining wounds. Device related adverse events were rare, limited to 0.8% inflammation in tunneled/undermined wounds. Infection occurred in 3.3% and 9.8%, and recurrence in 3.3% and 4.1%, respectively.

**Conclusions:** Findings show positive real‐world healing performance of FBD in wounds with complex geometry or drainage, with high closure rates and low adverse events. These data support its utility when standard dressings are less effective. Retrospective design and selection bias remain limitations, and prospective research is needed to confirm consistency across broader patient populations.

28

### 28.1 Bilayer Biodegradable Synthetic Matrix for Limb Preservation of Severe Foot Infections Post Surgical Debridement

#### 28.1.1 Nicholas Salerno, DPM; Marvin Mendez, RN, BSN; Anna Warner, RN

**Background:** Management and reconstruction of a surgically debrided foot can be complicated by infection risk and exposed avascular structures. Bilayer Biodegradable Synthetic Matrix (BBSM) can overcome those barriers in the comorbid patient population [1]. This case series describes BBSM use for coverage of bone and tendon while helping form a wound bed suitable for split‐thickness skin grafting (STSG) to preserve limb length after a severe foot infection (FI).

**Methods:** A retrospective analysis examined patients with severe FIs requiring surgical debridement and BBSM placement for bone and tendon coverage in 2025. Data collection included demographics, comorbidities, time from BBSM application to complete granulation tissue formation (CGTF), infection with BBSM, preserved limb length and number of BBSM applications.

**Results:** Four patients were identified with an even distribution of male and female and age range of 36 to 64. 75% had peripheral artery disease (PAD) and 50% had type 2 diabetes (T2D). Time from BBSM application to CGTF ranged from 27 to 55 days with no BBSM infections. 75% had one BBSM application and 25% had two. 75% received a STSG with complete graft take and 25% were lost to follow up after CGTF. 75% had preserved limb length with BBSM post amputation and 25% had limb salvage of the entire foot.

**Conclusions:** BBSM provided coverage of avascular structures and avoided infection while new tissue formed in one to two applications. Limb preservation of an infected foot is possible in this population with an extensive surgical debridement, BBSM and a STSG.

29

### 29.1 Flowable Porcine Urinary Bladder Matrix Complex Wounds: A Multicenter Prospective Study

#### 29.1.1 Malachy Asuku, MD, MBA; Jessica Evans; Michael Cripps, MD; Jeffrey Shupp, MD, FACS; Alysha Oropallo, MD, FACS

**Background:** Tunneling and undermining in chronic wounds are linked to inflammation, infection risk, and delayed healing, driving chronicity. Surgical excision may be effective but often impractical due to recurrence and anatomic limits. Porcine urinary bladder matrix (UBM) supports a pro‐remodeling environment and immune response. Flowable UBM facilitates precise delivery into tunnels and cavities. This multicenter prospective study evaluated its safety, effectiveness, and impact on overlying wound closure.

**Methods:** Following IRB approval and informed consent, enrolled subjects received flowable UBM applied to undermining and tunneling wound aspects and a combination of the particulate and sheet configuration of UBM to the overlying wound. The primary outcome was relative percentage reduction in tunneling volume or undermining depth at 12 weeks. Safety was assessed by device‐related adverse events.

**Results:** Of 25 enrolled subjects, 21 met per‐protocol criteria;15 (71%) had wounds exhibiting undermining and 6 (29%) had tunneling. At 12 weeks, complete resolution occurred in 11 wound aspects (52.4%), including 8/15 (53%) undermining and 3/6 (50%) tunneling aspects. Complete resolution occurred in 9/11 (81%) non–pressure ulcers and 2/10 (20%) pressure ulcers. Mean percentage reduction in tunneling/undermining dimensions was 50 ± 133% (median: 100%). No device‐related adverse events were reported.

**Conclusions:** This study demonstrated that flowable UBM facilitated the resolution of tunneling and undermining features in complex and difficult‐to‐heal wounds, supporting improved wound closure, overall healing, and potential limb salvage outcomes.

30

### 30.1 The Charcot–Gout–Infection Triad: An Under‐recognized Pathway to Limb Loss

#### 30.1.1 Courteney Asase, DPM; Alyson Boudreau, DPM; Caitlin Zarick, DPM

**Background:** Necrotizing soft‐tissue infections of the foot are limb‐ and life‐threatening emergencies that may be difficult to recognize in patients with neuropathy and chronic inflammatory foot disease. Although Charcot neuroarthropathy and gout are individually known to mimic infection, their coexistence with necrotizing infection and severe osseous destruction is rarely reported and poorly characterized.

**Methods:** We report two cases of a neuropathic foot presenting with a gas‐forming infection in the setting of advanced Charcot neuroarthropathy and extensive intraosseous gouty tophi. Intraoperative exploration revealed diffuse joint collapse with friable, “crumbly” bone involving multiple joints, as well as gross tophaceous deposits within bone and surrounding soft tissues.

**Results:** Patient 1 demonstrated systemic inflammation with imaging concerning for gas infection, Charcot, and interosseous gout. Intraoperatively, diffuse necrotizing infection with communicating wounds, copious purulence, and liquefied, friable midfoot bone containing gouty tophi was found, needing BKA. Patient 2 had multifocal infection with Charcot and localized tophi. Staged debridements with adjunctive revascularization and local antibiotics allowed limb salvage.

**Conclusions:** Charcot neuroarthropathy and gout do not merely mimic infection, they obscure necrotizing infection. In neuropathic feet, warmth, erythema, and swelling may reflect baseline Charcot, but painless. Gout flares elevate inflammatory markers without leukocytosis. Imaging may already show joint destruction or marrow edema, and gas may be misattributed to ulcer contamination, delaying diagnosis. Charcot and gout should heighten suspicion and prompt earlier surgical exploration to prevent limb loss.

31

### 31.1 Use of Topical Oxygen Therapy for Chronic Lower Extremity Wounds

#### 31.1.1 Hannah Soltani, BA; Ryan Houlihan; Abigail Anderson, DPM; Alyson Boudreau, DPM; John Steinberg, DPM; Christopher Attinger, MD

**Background:** Topical wound oxygen (TWO2) therapy has emerged as a treatment option for chronic lower extremity (LE) wounds due to its multimodal mechanism of action and ability to be administered in the home setting. This study evaluated wound size reduction in patients with diabetic foot ulcers treated with home‐based TWO2 therapy.

**Methods:** We conducted a retrospective review of 20 patients (with 27 wounds) treated at a multidisciplinary limb salvage center between September 2023 and December 2025 who underwent therapy with the TWO2 system (HyperBox; AOTI Ltd., Galway, Ireland). An oxygen–inflated extremity chamber was prescribed for 90 minutes per day, 5 days per week. The primary outcome was percent wound size reduction over the treatment period.

**Results:** Mean age was 69.9 ± 15.5 years, 85% were diabetic, and 45% had peripheral vascular disease. Wounds were most commonly at the ankle (55%), with a mean initial wound size of 25.3 ± 31.5 cm^2^, and 52% of the wounds were at the fascial level. Mean treatment duration was 247.2 ± 158.1 days. At final follow‐up, mean wound size decreased to 9.1 ± 17.0 cm^2^, corresponding to an average wound size reduction of 80.5% ± 27.7%. Six patients (30%) had re‐ulceration, and one patient progressed to BKA.

**Conclusions:** TWO2 therapy is associated with substantial wound size reduction in a high‐risk population with chronic LE wounds refractory to traditional treatments, supporting its role as a limb salvage adjunct in select patients.

32

### 32.1 Female Sex as a Predictor of Early Mortality After Free Tissue Transfer for Limb Salvage: A Single Institution's Experience

#### 32.1.1 Hannah Soltani, BA; Christopher Ply, BS; Rachel Rohrich, BS; Cameron Akbari, MD; Richard Youn, MD; Christopher Attinger, MD; Karen Evans, MD

**Background:** Free tissue transfer (FTT) is used for reconstructing chronic non‐healing lower extremity (LE) wounds in high‐risk patients. Identifying early mortality risk factors is critical for patient counseling and perioperative care. This study evaluates overall survival and predictors of death within one year.

**Methods:** We retrospectively reviewed 396 patients undergoing LE FTT (median follow‐up 16.6 [IQR: 27.2] months). Survival was measured from FTT to death or last follow‐up. Kaplan‐Meier analysis estimated survival probability. Early death was defined as mortality within one year. Firth logistic regression evaluated associations with patient and surgical factors.

**Results:** Early mortality was associated with higher Charlson Comorbidity Index (OR 1.41 per point; p=0.045). Diabetes showed a nonsignificant trend toward increased risk. Female sex compared to male had a 5.73 times higher odds of early death. (OR 5.73; 95% CI 1.43–22.98; p=0.014). Kaplan‐Meier analysis demonstrated significantly lower 1‐year survival among females compared with males (log‐rank p = 0.01). At 12 months, survival was approximately 75% for males and 55% for females.

**Conclusions:** Female sex is an independent predictor of early mortality after LE FTT. These findings underscore the need for sex‐specific risk stratification and further study of disparities in limb salvage outcomes.

33

### 33.1 A 14.5‐year Experience Of Free Tissue Transfer For Chronic Lower Extremity Wounds: A Focus On Vasculoplastic Approaches Improving Limb Salvage And Patient‐Reported Outcomes From 417 Flaps At A Tertiary Wound Care Center

#### 33.1.1 Hannah Soltani, BA, Christopher M. Ply, BS, Leila I. Jones, BS, Christopher E. Attinger, MD, Richard C. Youn, MD, Cameron Akbari, MD, Karen K. Evans, MD

**Background:** Free tissue transfer (FTT) remains a cornerstone of multidisciplinary management for chronic lower extremity (LE) wounds. We characterized long‐term outcomes and patient‐reported functional recovery in a complex patient population.

**Methods:** We retrospectively reviewed 417 LE FTTs performed from July 2011 to October 2025. Demographics, comorbidities, vascular status, flap characteristics, and postoperative outcomes were analyzed. A subset of patients completed validated patient‐reported outcome measures (PROMs), including the Lower Extremity Functional Scale (LEFS), PROMIS‐3a, SF‐12, and SRQ‐20 at regular time intervals.

**Results:** Mean age was 56.8 ± 13.7 years and BMI was 29.9 ± 6.5 kg/m^2^. Comorbidities commonly included diabetes (55%) and neuropathy (42%). Most wounds involved the ankle (35%) or hindfoot (23%), with median wound area of 75 cm^2^ [IQR 77.8], and 18% requiring preoperative revascularization. Flap success was 97%, with a 5% takeback rate and 88% limb salvage at follow‐up. Median time to ambulation was 2.7 months [IQR 5.0]. Mean LEFS improved from 37.8 ± 8.9 (47% function) to 46.8 ± 23.4 (58% function).

**Conclusions:** This large single‐institution series demonstrates successful limb salvage with high functional recovery and pain improvement in a medically complex population undergoing LE FTT.

34

### 34.1 “If You’re Not Walking, You’re Dying”: Survival And Ambulation After Functional Lower Extremity Reconstruction

#### 34.1.1 Hannah Soltani, BA; Christopher Ply; Rachel Rohrich, BS; Richard Youn, MD; Christopher Attinger, MD; Karen Kim Evans, MD

**Background:** Ambulation after lower extremity (LE) reconstruction represents more than functional recovery: it may be a marker of survival. While technical success in limb salvage is often prioritized, the long‐term outcomes of non‐ambulatory patients remain unclear. This study investigates the relationship between postoperative ambulation and mortality following nontraumatic LE reconstruction.

**Methods:** A retrospective review was performed of 745 patients undergoing LE reconstruction with local flaps, free tissue transfer (FTT), or below‐knee amputation (BKA) from 2011‐2023. Patients were stratified by ambulatory status at final follow‐up: ambulatory vs. non‐ambulatory (NA). Median follow‐up was 25.6 months [IQR: 43.5]. Multivariate logistic regression identified predictors of mortality.

**Results:** At final follow‐up, 511 patients (68.6%) were ambulatory. NA patients had higher rates of end‐stage renal disease (34.6% vs. 17.6%) diabetes (81.2% vs. 63.0%) and coronary artery disease (34.2% vs. 17.6%, all p<0.001. Preoperative ambulation was highest in FTT (87.7%), followed by local flaps (84.7%) and BKA (79.4%). Ambulatory patients had 41% lower odds of death (OR 0.59, 95% CI 0.41‐0.85, p=0.006). One‐year mortality was significantly higher in NA patients (12.4% vs. 2.7%, p<0.001).

**Conclusions:** Ambulation is a strong independent predictor of mortality, highlighting the life‐saving importance of restoring mobility. Therefore, our limb reconstruction algorithm and techniques prioritize function first.

35

### 35.1 When Limb Salvage Fails: Identifying Predictors Of Early Amputation After Microsurgically Successful Free Flap Reconstruction

#### 35.1.1 Hannah Soltani, BA; Christopher Ply, BS; Leila Jones, BS; Rachel Rohrich, BS; Richard Youn, MD; Christopher Attinger, MD; Cameron Akbari, MD; Karen Kim Evans, MD

**Background:** Free tissue transfer (FTT) is a common reconstructive method for limb salvage of complex lower extremity (LE) wounds. However, a subset of patients undergo major amputation within six months despite technically successful reconstruction, representing failed limb salvage. This study aims to identify predictors of early amputation and characterize this high‐risk group.

**Methods:** We conducted a retrospective review of 409 LE FTTs performed from 2011‐2024. Patients were stratified by early amputation status (<6 months). Comorbidities, vascular imaging, wound characteristics, and postoperative outcomes were compared. Firth logistic regression identified independent predictors of early amputation and evaluated a composite high‐risk cluster including high medial arterial calcification (MAC) score, end‐stage renal disease, positive cultures, and single‐vessel runoff.

**Results:** 22 patients (5.4%) underwent early amputation. Early amputation was associated with chronic steroid use (18% vs 6%, p=0.043), wound location at the midfoot (36.4% vs 17.3%, p=0.025), venous reflux (p=0.011), any postoperative wound complication (77% vs 29%, p<0.001), and infection (59% vs 13%, p<0.001). In multivariable modeling, the composite high‐risk cluster was associated with greater odds of early amputation (OR 2.8, p=0.054).

**Conclusions:** Early amputation after FTT is a failure and should be better studied and reported. This reflects a multifactorial process involving vascular compromise, metabolic disease, and recurrent infection. Preoperative identification of this high‐risk cohort may refine patient selection and optimize limb salvage strategies.

36

### 36.1 AI‐Based Assessment of Tissue Characteristics in Diabetic Foot Ulcers: Integrating Objective Wound Metrics for Reconstructive Planning

#### 36.1.1 Nichole Andrade; Hannah Soltani, BA; Richard Youn, MD; Christopher Attinger, MD; Kenneth Fan, MD; Karen Kim Evans, MD

**Background:** Diabetic foot ulcers (DFUs) precede up to 85% of lower extremity amputations and affect nearly one in four patients with diabetes, with recurrence rates up to 80%. Existing DFU assessment scales are subjective and fail to capture variables relevant to reconstructive surgery. We developed a deep learning framework to assess DFUs using objective wound metrics.

**Methods:** A dataset of 110 DFU images was used to develop a convolutional neural network (CNN) capable of delineating wound tissues and deriving novel DFU assessment variables operationalized for artificial intelligence (AI) training. Each variable was quantitatively defined and mapped to reconstructive significance, capturing features relevant to vascularity, wound size, border complexity, and tissue viability.

**Results:** The model achieved good performance for wound tissue delineation, with a mean Dice of 80.3% and IOU of 71.7% for granulation, fibrin and callus segmentation. AI‐derived reconstructive features strongly correlated with reference standards: wound area (R=0.98), granulation (0.97), fibrin (0.90), callus (0.98), perimeter (0.86), circularity (0.87), solidity (0.89), aspect ratio (0.97), callus ring fraction (0.96), Lab a* (0.99), fractional area (0.98), peri‐ΔE (0.96), and mean depth (0.91).

**Conclusions:** This first‐of‐its‐kind model validated DFU assessment variables tailored to reconstructive planning with strong technical performance. By aligning algorithm design with surgical context, it establishes a foundation for AI integration into limb salvage.

37

### 37.1 Predictors of Split Thickness Skin Graft Failure in Chronic LE Wounds: Our 10‐Year Experience

#### 37.1.1 Celina Zhao; Hannah Soltani, BA; Ryan Houlihan, BS; Jayson Atves, DPM; John Steinberg, DPM; Richard Youn, MD; Christopher Attinger, MD; Karen Kim Evans, MD

**Background:** Split‐thickness skin grafting (STSG) remains a widely used management strategy for chronic lower extremity (LE) wounds, yet failure is common, particularly in highly comorbid patients. This study evaluates independent risk factors associated with STSG failure in chronic LE wounds in a highly comorbid population.

**Methods:** A retrospective chart review of patients undergoing STSG for chronic LE wounds between January 2015 and December 2024 was conducted. Patient demographics, comorbidities, wound characteristics, culture results, and STSG factors were collected. The primary outcome was graft failure, defined as <80% graft take at final follow‐up. Univariate and multivariable logistic regression analyses were performed to identify independent predictors of failure.

**Results:** A total of 477 wounds were included from 334 unique patients, with an overall graft failure rate of 17.2%. Smoking history was significantly associated with unsuccessful STSG outcomes (X2 =8.30, p=0.0158). Smokers also demonstrated approximately 60% lower odds of graft success compared with never‐smokers (OR=0.41, p=0.0062). In terms of wound location, leg wounds had a significant association with unhealed wounds (X2 =3.93, p=0.04746). Leg and hindfoot wounds were approximately 50% less likely to achieve successful graft healing compared with other LE locations (OR=0.51, p=0.0425; OR=0.47, p=0.0469). Patients who received postoperative negative pressure wound therapy (NPWT) had 86% higher odds of STSG success compared with those who received traditional or bolster dressings (OR=1.86, p=0.0419).

**Conclusions:** Identification of high‐risk features, including smoking and wound location, may guide patient selection and wound bed optimization strategies, while postoperative NPWT represents a potentially modifiable factor associated with improved STSG success.

38

### 38.1 Machine Learning–Based Prediction of Lower Extremity Amputation in Patients with End Stage Renal Disease on Hemodialysis

#### 38.1.1 Nichole Andrade; Hannah Soltani, BA; Cameron Akbari, MD; Kenneth Fan, MD; Richard Youn, MD; Christopher Attinger, MD; Karen Kim Evans, MD

**Background:** Patients with end stage renal disease on hemodialysis face disproportionately high lower extremity amputation risk driven by compounded ischemic, metabolic, and vascular factors. Accurate risk stratification requires multivariable modeling and close surveillance of those at highest limb loss risk. We aimed to develop machine learning–based predictive models to identify key risk factors and enable early screening of high risk ESRD patients.

**Methods:** A retrospective multicenter study of 875 patients with ESRD on hemodialysis from January 2012 to November 2022 across four tertiary hospitals was conducted. Demographic, vascular, metabolic, and functional variables were extracted to develop and train two ML models predicting LE amputation. Performance was assessed using ROC AUC, sensitivity, specificity, and calibration (Brier score). Feature importance was evaluated with SHAP and multivariable logistic regression.

**Results:** The regression/classification model achieved AUC 0.874 and 90% accuracy, demonstrating strong discrimination for LEA, with 45% sensitivity and 95% specificity. The non linear classifier performed better, with 91% accuracy and AUC 0.911, sensitivity 55% and specificity 96%. Both showed excellent calibration (Brier <0.10). Vascular disease and prior foot wounds were dominant predictors (OR 3.00, p<0.001).

**Conclusions:** Both ML models demonstrated excellent discrimination for LE amputation (AUC 0.87–0.91), outperforming validated ESRD risk models (C index 0.76–0.8, Akerboom 2024), though external validation is needed. Feature attribution confirmed multifactorial risk, with prior foot wounds, peripheral arterial disease, and prior vascular intervention as dominant predictors. Integrating vascular, functional, and comorbidity data within ML supports precision risk stratification and proactive limb salvage.

39

### 39.1 Free Flap Limb Salvage For Chronic Lower Extremity Wounds: Does Gender Influence Outcomes?

#### 39.1.1 Hannah Soltani, BA; Christopher M. Ply, BS; Alexandra Junn, MD; Christopher E. Attinger, MD; Richard C. Youn, MD; Cameron Akbari, MD; Karen K. Evans, MD

**Background:** Sex differences in presentation, treatment, and outcomes are well described in peripheral arterial disease (PAD) and chronic limb‐threatening ischemia. Whether such disparities exist in patients undergoing free flap reconstruction for atraumatic, chronic, non‐healing lower extremity wounds is unknown.

**Methods:** We retrospectively reviewed 409 consecutive free flap reconstructions for limb salvage (290 male, 119 female). Demographics, comorbidities, wound characteristics, vascular imaging, intraoperative flap selection, and postoperative outcomes were compared by sex. Multivariable logistic regression was used to examine associations between sex and flap success, adjusting for comorbidities, wound size, flap type, and vascular status.

**Results:** Men had higher rates of diabetes (59% vs 43%, p=0.003) and higher HbA1c (6.4% vs 5.9%, p=0.003), while women had lower hemoglobin (9.3 vs 9.7 g/dL, p=0.008) and smaller wounds (58 vs 80 cm^2^, p=0.018). Early flap success was higher in men (98.6% vs 92.4%, p=0.007). After adjustment, sex was not independently associated with flap success (OR 0.44, 95% CI: 0.09‐2.11, p=0.31). Post hoc analysis demonstrated adequate power (88%) to detect moderate differences between groups.

**Conclusions:** Although sex‐specific differences in comorbidities exist, free flap outcomes were equivalent between men and women. These findings suggest that our standardized multidisciplinary limb salvage protocols may mitigate the sex‐based disparities reported in broader PAD populations.

40

### 40.1 Computer Vision for Wound Care: A Multitask Deep Learning System for Dressing Recommendation

#### 40.1.1 Nichole Andrade; Hannah Soltani, BA; Richard Youn, MD; Christopher Attinger, MD; Kenneth Fan, MD; Karen Kim Evans, MD

**Background:** Wound dressing selection is fundamental yet highly variable, shaped by etiology, exudate burden, and clinician preference. Prior computer vision work has focused on wound segmentation or classification, but few systems translate image‐based assessment into actionable treatment guidance. We developed and evaluated a multitask deep learning decision‐support system integrating segmentation and classification to generate clinically relevant dressing recommendations.

**Methods:** A dataset of 1,037 wound images with segmentation masks was annotated for wound type, state, and dressing choice. A multitask CNN was trained for joint segmentation and eight‐class etiologic classification of wounds. Performance was evaluated on held‐out data using accuracy and Dice. Dressing recommendations were generated using an evidence‐based rule framework mapping predicted features to absorptive, moisture‐donating, or protective categories. Agreement with clinician labels was assessed.

**Results:** Our multitask deep learning model demonstrated strong wound understanding, achieving a wound‐type classification accuracy of 80.6% and a segmentation Dice score of 0.61 on the validation set. Performance for dressing recommendations was robust, evaluated using clinical functional categories, with the system achieving 78.6% agreement with evidence‐based recommendations. Performance for absorptive dressings was highest, with an F1‐score of 0.88.

**Conclusions:** This work presents a clinically aligned computer vision framework that moves beyond wound identification to support treatment decisions. By coupling automated segmentation and classification with functional dressing recommendations, the system demonstrates strong performance in guiding a key aspect of wound care. This approach emphasizes clinical utility, offering a scalable foundation for AI‐assisted wound management and a meaningful step toward actionable computer vision in surgical practice

41

### 41.1 Anemia Screening in a Surgical Wound Clinic Using Transcutaneous Hemoglobin Monitoring

#### 41.1.1 Cameron Darnell, BA; Hannah Soltani, BA; Christopher Ply, BS; Ryan Houlihan, BS; John Steinberg, DPM; Jayson Atves, DPM; Russell Wall, MD; Richard Youn, MD; Christopher Attinger, MD; Karen Kim Evans, MD

**Background:** Anemia negatively impacts wound healing, graft take, oxygen delivery, and post‐operative recovery. Despite this, routine non‐invasive anemia screening is not standard practice in outpatient wound clinics. Delayed identification of iron‐deficient anemia may contribute to longer healing times, increased complication rates, and preventable blood transfusions.

**Methods:** In an ongoing prospective study beginning on November 3, 2025, we evaluated the feasibility of transcutaneous hemoglobin (Hb) screening using the Masimo Rad‐67 device in a high‐risk lower extremity limb salvage population seen at the Center for Wound Healing (CWH) at MGUH. Both preoperative and postoperative patients were screened. Hemoglobin values lower than 10.5 g/dL were considered anemic and in need of a confirmatory blood test (CBC, ferritin, TIBC, serum iron, iron saturation).

**Results:** A total of 141 patients were screened, all of whom were surgical. 90% (127/141) of patients were screened postoperatively, and 10% (14/141) were screened preoperatively. Anemia (Hb <10.5 g/dL) was identified in 8.5% (12/141) of all patients, all of whom fell into the postoperative category, whose last date of surgery was between October 2025 and January 2026. Ten out of twelve of those surgeries had been performed on lower extremities. Mean patient age was 61 years, with 67% M and 32% F.

**Conclusions:** Anemia is relatively prevalent in our surgical, high‐risk wound care population. We propose the feasibility of a simple algorithm for increased point‐of‐care monitoring via noninvasive testing and a streamlined referral process for confirmatory iron‐specific anemia testing and prompt intravenous management.

42

### 42.1 Transmetatarsal Amputation vs. First Ray Amputation: Comparative Outcomes in Diabetic Limb Salvage

#### 42.1.1 Robert Chubb, DPM; Timmer Hoffmeister, DPM; Christopher Ply; Christopher Attinger, MD; John Steinberg, DPM

**Background:** First ray amputation and transmetatarsal amputation (TMA) are common limb salvage procedures for diabetic foot infection. However, both operations have been associated with high rates of wound complications, biomechanical imbalance, and progression to more proximal amputation, raising concerns about long term durability and function. This study evaluates clinical outcomes following first ray amputation versus TMA to assess the validity of first ray resection as a limb‐preserving strategy.

**Methods:** A retrospective review was performed using the Georgetown University Hospital Limb Salvage Database. Patients who underwent first ray amputation or transmetatarsal amputation were identified by CPT coding. Demographics, wound complications, ulcer recurrence, revisional surgery, progression to proximal amputation, and adjunctive procedures were analyzed and compared between cohorts.

**Results:** A total of 74 patients were included (50 TMAs, 24 first ray amputations). There were no statistically significant differences between groups in wound dehiscence (14% TMA vs. 8.3% first ray), ulcer recurrence (22% TMA vs. 58.3% first ray), need for revisional surgery (38% TMA vs. 41.7% first ray), or progression to proximal amputation (2% TMA vs. 12.5% first ray). The TMA cohort underwent significantly more concomitant tendon balancing procedures (60%) compared with the first ray cohort (16.7%).

**Conclusions:** Despite concerns regarding biomechanical instability and ulcer recurrence, first ray amputation demonstrated comparable overall complication and limb preservation rates to TMA in this cohort. These findings support first ray amputation as a viable limb salvage option in appropriately selected patients. Further prospective studies are warranted to clarify functional outcomes and optimize surgical decision making in partial foot amputations for diabetic limb salvage.

43

### 43.1 Preliminary Outcomes of a Patient Navigator Initiative in a Multidisciplinary Wound Clinic

#### 43.1.1 Cameron Darnell, BA; Hannah Soltani, BA; Christopher Ply, BS; John Steinberg, DPM; Jayson Atves, DPM; Richard Youn, MD; Christopher Attinger, MD; Karen Kim Evans, MD

**Background:** Patient navigation can be an effective way to improve healthcare outcomes and provide meaningful patient support. The patient navigator role consists of regular contact with patients, including facilitating primary care provider (PCP) appointments, coordinating specialist referrals, connecting patients to transportation resources, providing educational materials regarding diabetes management or nutrition, and providing structured follow‐up.

**Methods:** This is a retrospective descriptive analysis of patients seen at the Center for Wound Healing (CWH) at MGUH from November 2025 to February 2026 who utilized the patient navigator pathway (PNP). Variables analyzed included referral reason, presence of a PCP, social barriers (including home support and transportation), and navigation actions performed (including PCP referral, transportation coordination, and specialist/education referral). A total of 16 patients were involved in the PNP.

**Results:** The most common referral reasons for this cohort included lack of established PCP (8/16, 50%), transportation barriers (2/16, 12.5%), difficulty coordinating specialty care (3/16, 19%), assistance in obtaining surgical clearance (2/16, 12.5%), and insurance or follow‐up challenges (3/16, 19%). Around 75% (12/16) of patients utilized the PNP for assistance in establishing or expediting a PCP. 50% (8/16) utilized the PNP for coordinating postoperative follow‐up and nursing/living support.

**Conclusions:** Surgical patient navigation can provide support for transportation insecurity, difficulty attending appointments, and challenges navigating health systems. We demonstrate feasibility for patient navigator implementation and will continue to assess how this role impacts patient follow‐up, compliance, and outcomes.

44

### 44.1 Four Revascularization Attempts of Small Vessel Disease Across Three Institutions: Advocating for Patient Preference in Limb Salvage

#### 44.1.1 Sana Siddiqui, DPM, MS; Jaclyn Wessinger

**Background:** Severe infrapopliteal arterial disease presents significant challenges to limb salvage in patients with diabetes, particularly when distal runoff is limited. We present a case highlighting the role of vascular surgery persistence, multidisciplinary coordination, and patient advocacy in preventing major amputation after multiple prior failed revascularization attempts across separate institutions.

**Methods:** A 76‐year‐old male with type 2 diabetes mellitus, tobacco use, cardiovascular disease with implanted cardioverter‐defibrillator, and prior intracranial hemorrhage presented with progressive left hallux erythema and ischemic pain. Computed tomographic angiography demonstrated severe multilevel peripheral arterial disease. After three prior endovascular procedures at outside institutions, distal revascularization was deemed unfeasible and major amputation was recommended.

**Results:** The patient declined amputation and underwent a fourth endovascular attempt using selective pedal access. Angioplasty of the SFA, popliteal, tibioperoneal trunk, and anterior tibial arteries restored single‐vessel runoff via the anterior tibial artery. Immediate coordinated podiatric intervention involved hallux amputation. Postoperatively, the patient demonstrated improved perfusion, viable flaps, wound healing, and reduced ischemic pain. Vascular studies demonstrated improved waveforms.

**Conclusions:** This case underscores the importance of persistent revascularization efforts, multidisciplinary collaboration, and precise surgical timing in achieving limb salvage despite severe diabetic PAD with limited distal runoff.

45

### 45.1 Percutaneous Endovascular Intervention and Free Tissue Transfer Provides Durable and Functional Limb Salvage in High‐Risk Patients: Updated Outcomes of 413 Lower Extremity Reconstructions Over 14 Years

#### 45.1.1 Hannah Soltani, BA; Rachel Rohrich, BS; Christopher Ply, BS; Karen R. Li, MD; Elonay Yehualashet, BS; Christopher Attinger, MD; Richard Youn, MD; Cameron M. Akbari, MD; Karen Kim Evans, MD

**Background:** We have demonstrated the role of lower extremity (LE) free tissue transfer (FTT) among highly comorbid patients with chronic non‐healing wounds, and the importance of preoperative arteriography for reconstructive success. While percutaneous endovascular intervention (PEI) is well established for limb salvage, its impact prior to FTT on functional recovery remains incompletely characterized. We evaluate long‐term outcomes of LE FTT with preoperative PEI using our vasculoplastic approach.

**Methods:** A retrospective review was conducted of all LE FTT performed for limb salvage at a single institution between July 2011 and August 2025. Patients were stratified based on receipt of preoperative PEI: Group 1 (PEI) and Group 2 (no PEI). A total of 413 LE FTTs were performed, of which 77 (18.6%) underwent preoperative PEI. Comorbidities (including diabetes mellitus [DM] and chronic kidney disease [CKD]), wound and vascular characteristics, and long‐term functional outcomes were analyzed.

**Results:** Group 1 patients were older (median 60 vs 57 years, p=0.025) and had higher rates of DM (88.3% vs 47.0%, p<0.001) and CKD (27.3% vs 9.2%, p<0.001). At a median follow‐up of 12.2 (IQR: 24.1) months, limb salvage (87.7%), ambulation (82.7%), and mortality (6.1%) rates were comparable between the two groups. Among the entire cohort, 65 patients (15.7%) underwent post‐operative arteriograms due to slow healing of the FTT, of which a higher proportion were in Group 1 (42.9% vs 9.5%, p<0.001)

**Conclusions:** FTT in conjunction with PEI performed within a multidisciplinary vasculoplastic approach provides successful and durable limb salvage, functional recovery, and ambulation in patients with multiple severe comorbidities.

46

### 46.1 Factors Contributing to Prolonged Hospital Stay in Lower Extremity Free Flap Patients: A Retrospective Data Analysis

#### 46.1.1 Christopher Ply; Alexandra Junn, MD; Hannah Soltani, BA; Sarah Kanner, BS; Richard Youn, MD; Christopher Attinger, MD; Cameron Akbari, MD; Stephen Baker, MD; Karen Kim Evans, MD

**Background:** Length of stay (LOS) is an increasingly important metric, and we aim to identify factors associated with extended LOS in lower extremity (LE) free tissue transfer (FTT) patients to distinguish delays due to medical complexity versus discharge processes.

**Methods:** A retrospective review of the last 101 LE FTTs at a single tertiary center from 2023 to 2025 was performed. Prolonged LOS was =75th percentile of the median. Initial physical therapy (PT) evaluation after final surgery was used as a proxy for medical stability to compare time for medical versus discharge care. Logistic regression assessed predictors of prolonged LOS.

**Results:** Median LOS was 27 [IQR: 21] days, with 20 [IQR: 18] days towards medical care and 8 [IQR:7] days towards discharge care. Prolonged LOS was =41 days and associated with higher Charlson Comorbidity Index scores (6 [4] vs 4 [4], p=0.017). Most (52.5%) patients in the prolonged LOS group were sent to acute rehab (p=0.003). Each 1 cm^2^ increase in wound size increased odds of prolonged LOS by 0.8% (OR = 1.008, p < 0.05). Dialysis was associated with 13 additional hospitalization days (OR=13.1, p=0.050).

**Conclusions:** Medical complexity contributes more towards prolonged length of stay. Targeting perioperative optimization may reduce LOS while maintaining quality of care.

47

### 47.1 Predicting the Need for Vascular Surveillance After Lower Extremity Free Tissue Transfer for Limb Salvage: An Updated Analysis

#### 47.1.1 Hannah Soltani, BA; Rachel N. Rohrich, BS; Christopher Ply, BS; Karen R. Li, MD; Sabrina Deleonibus, BS; Jie Jung Shih, BS; Kishan Shah, BS; Christopher E. Attinger, MD; Richard C. Youn, MD; Cameron M. Akbari, MD; Karen K. Evans, MD

**Background:** Vascular evaluation and optimization are fundamental to lower extremity (LE) free tissue transfer (FTT) success. We have previously shown the durability and success of percutaneous endovascular intervention prior to LE FTT, but there exists a lack of consensus regarding postoperative surveillance. Therefore, we have studied a large cohort of LE FTT patients undergoing postoperative revascularization (PR), to define an appropriate surveillance strategy.

**Methods:** A retrospective review was conducted of all LE FTTs performed for limb salvage at a single institution from July 2011‐June 2025. All patients underwent preoperative diagnostic arteriography, with endovascular intervention performed as indicated. Patients were stratified based on receipt of PR or No PR during follow‐up. Comorbidities, wound and vascular characteristics, and long‐term outcomes were analyzed. Multivariable logistic regression was used to identify independent predictors of PR.

**Results:** Of 369 LE FTTs, 34 patients (9.2%) required PR at a median follow‐up of 17 months. Patients requiring PR had increased rates of diabetes (97.0% vs. 51.9%, p<0.001), peripheral arterial disease (96.7% vs. 50.3%, p<0.001), chronic kidney disease (32.4% vs 10.4%, p<0.001), and end‐stage renal disease (17.6% vs 5.1%, p=0.004). Prior preoperative vascular intervention (OR 5.0, 95% CI 1.7–15.0, p=0.001) and osteomyelitis at reconstruction (OR 3.4, 95% CI 1.1–10.5, p=0.039) independently predicted PR.

**Conclusions:** Patients with extensive comorbidities and LE atherosclerosis requiring vascular intervention prior to FTT mandate a close postoperative vascular surveillance by a team of both vascular and plastic surgery specialists to achieve durable limb salvage.

48

### 48.1 Optimizing the Use of a Chimeric Anterolateral Thigh Adipofascial Free Flap for Coverage of Recipient Site Anastomosis

#### 48.1.1 Karen R. Li, MD, Hannah Soltani, BA; Rachel N. Rohrich, BS; Anusha Singh, MD, Ryan P. Lin, MD; Sami Ferdousian, MS; Christopher E. Attinger, MD; Richard C. Youn, MD; Karen K. Evans, MD

**Background:** Tension‐free closure over the vascular pedicle is essential to flap survival, but is difficult in chronic lower extremity wounds, where recipient vessels often require long pedicles. Attempting primary closure can compromise flow as tissue is often indurated, inelastic, and poorly vascularized. Therefore, we developed a chimeric anterolateral thigh (ALT) adipofascial flap combining a muscle component for defect coverage and a vascularized fascial component to protect the micro‐anastomosis.

**Methods:** We retrospectively reviewed all patients who underwent lower extremity free tissue transfer using this flap design by the senior author between 2022 and 2023. The chimeric design consisted of a proximally based vastus lateralis muscle flap for primary defect coverage and a separate, thin adipofascial flap, both perfused by independent perforators from the descending branch of the lateral circumflex femoral artery, used to provide tension‐free coverage of the microvascular anastomosis.

**Results:** Ten patients (mean age, 60.4 ± 8.1 years; mean Charlson Comorbidity Index, 4.7 ± 2.3) underwent reconstruction of LE wounds averaging 91.9 ± 76.7 cm2. All flaps survived without requiring takeback. Patients were followed for 8.1 ± 5.6 months. One patient ultimately underwent below‐knee amputation 396 days following the index FTT.

**Conclusions:** The chimeric ALT adipofascial flap provides reliable, tension‐free coverage of both the defect as well as the pedicle and microanastomosis when primary closure is not feasible. Early experience demonstrates 100% flap survival and 88.9% limb salvage in a high‐risk cohort.

49

### 49.1 Patient‐Reported Outcomes and Functional Consequences of Ray Amputations: An Evidence‐ Based and PROM‐Driven Approach for Functional Limb Salvage within the Forefoot

#### 49.1.1 Nori Pham, MD, PhD, MS

**Background:** The pedal rays play a critical role in normal ambulation, and their loss via amputation has been associated with poor function, reduced quality of life, and increased morbidity. As a result, the transmetatarsal amputation (TMA) has been cited as a more predictable, functional, and superior amputation level. We seek to better understand and characterize the consequences of the poorly understood ray amputation in comparison to the TMA.

**Methods:** A single‐center retrospective review of adult patients undergoing TMA and pedal ray amputations from June 2021 to June 2023 was performed. Patients were stratified into cohorts based on amputation type. Outcomes were defined as major and minor complications, rates of limb salvage, and patient‐reported function and quality of life using the Lower Extremity Functioning Scale (LEFS) and 12‐item short‐form (SF‐12), respectively.

**Results:** In this cohort, 95 amputees were identified: 50 (52.6%) underwent TMA and 45 (47.4%) ray amputation. Mean age was 64.6 ± 11.9 years with a Charlson Comorbidity Index of 5.4 ± 2.1. At median follow‐up of 12.1 months (IQR 24.1), limb salvage was 97.9% with 68.4% complications. Ray amputations had more minor non‐operative amputations (p<0.001), but similar major complications (p=0.81), function (LEFS 42.3±21.6 vs 44.7±17.1; p=0.590), and SF‐12 (30.2±4.6 vs 28.5±2.9; p=0.791).

**Conclusions:** When performed in the properly selected patient, TMA and ray amputation may not result in a significant difference in ultimate limb salvage, complication rate, or patient‐reported subjective function or quality of life outcomes.

50

### 50.1 Targeted Nerve Interventions in Highly Comorbid Through‐Knee Amputees: Advancing Palliative Care for Neuropathic Pain

#### 50.1.1 Christopher Ply, BS; Hannah Soltani, BA; Sami Ferdousian, MS; Danny Chamaa, MS; Ryan Bender, MD; Richard Youn, MD; Christopher Attinger, MD; Grant Kleiber, MD

**Background:** Through‐knee amputation (TKA) is an underutilized yet viable major lower extremity amputation level for both non‐ambulatory and ambulatory patients. A primary concern after amputation is pain. Targeted peripheral nerve interventions at the time of below‐ and above‐knee amputation have been shown to reduce neuropathic pain, but no study has exclusively assessed the outcomes of targeted peripheral nerve interventions performed at the time of TKA only.

**Methods:** A retrospective review was performed of all TKAs with primary targeted peripheral nerve intervention at a single institution between March 2018 and January 2024. Preoperative non‐ambulators were compared to preoperative ambulators. Primary outcomes included postoperative pain, narcotic, and neuromodulator usage at routine postoperative intervals.

**Results:** Thirty‐six TKAs were performed in 32 patients with a high comorbidity burden (mean Charlson Comorbidity Index 6.6 ± 3.4). TMR was performed in 72.2% of amputations, RPNI in 13.9%, and ETE coaptation in 13.9%. At a final follow‐up time of 13.5±9.8 months, 69.6% of limbs were pain‐free, with residual limb pain and phantom limb pain reported in 13.0% and 17.4%, respectively. Narcotic use declined significantly over time, with only 5.3% of patients requiring opioids at final follow‐up (p=0.014).

**Conclusions:** Targeted peripheral nerve interventions performed at the time of through‐knee amputation are safe and associated with low rates of chronic postoperative pain and long‐term narcotic dependence. These findings support the use of proactive nerve management at the TKA level and suggest that TKA with targeted nerve intervention is a viable option for non‐ambulatory and appropriately selected ambulatory patients.

51

### 51.1 Function‐Driven Algorithmic Approach to the Management of Heel Ulceration in the Chronic Wound Population: A Reconstructive Roadmap for Foot and Ankle Reconstructive Surgeons

#### 51.1.1 Christopher Ply, BS; Kishan Shah, BS; Luke Llaurado, BS; Hannah Soltani, BA; Rachel Rohrich, BS; Jayson Atves, DPM; John Steinberg, DPM; Cameron Akbari, MD; Richard Youn, MD; Karen K. Evans, MD; Christopher Attinger, MD

**Background:** Heel ulceration is a limb‐threatening condition associated with highest rates of ulcer recurrence and amputation risk among all foot ulcer locations. Differences in biomechanics and functional demands between plantar and posterior heel ulcers may influence treatment strategy and outcomes. We aim to compare the reconstructive approaches and clinical outcomes between plantar and posterior heel ulcers treated at a multidisciplinary limb salvage center.

**Methods:** A retrospective review was performed of 124 patients undergoing surgical management of heel ulceration. Patients were stratified by ulcer location (plantar vs posterior). Demographics, comorbidities, vascular status, operative interventions, and postoperative outcomes were analyzed. Primary outcomes included ambulation, re‐ulceration, progression to major lower extremity amputation, and mortality.

**Results:** Among 124 patients, 71 (57.3%) had plantar and 53 (42.7%) posterior heel ulcers. Posterior ulcer patients were older (64.9 vs 59.8 years, p=0.024), less ambulatory preoperatively (39.6% vs 88.7%, p=0.016), and had smaller wound areas (20.0 vs 40.0 cm^2^, p=0.020). Calcanectomy was performed in 64.5% of patients. Plantar ulcers were more commonly treated with local or free flaps and posterior ulcers with split‐thickness skin graft (p=0.004). Plantar ulcers demonstrated higher re‐ulceration rates (49.3% vs 18.9%, p=0.003).

**Conclusions:** Plantar heel ulcers exhibited substantially higher ambulation rates and re‐ulceration, emphasizing the importance of biomechanical correction and durable offloading in treatment planning. For posterior heel ulcers, offloading pressure is paramount for healing and preventing recurrence.

52

### 52.1 Biodegradable Bilayer Synthetic Matrix may be Appropriate to use on Wounds in patients who Have Undergone Deep Vein Arterialization

#### 52.1.1 Tara Robertson, BSN; Paul Kim, DPM, MS

**Background:** Peripheral artery disease affects 200 million people worldwide, with 11% of them experiencing chronic limb‐threatening ischemia (CLTI) associated with increased amputation and mortality (24% in the first year and 60% at 5 years). Deep vein arterialization (DVA) is for those without options to treat CLTI or without suitable vessels for revascularization. DVA uses a tibial artery‐to‐vein fistulization and other interventions, enabling retrograde delivery of oxygenated blood down a venous path. 1

**Methods:** A patient with diabetes and peripheral vascular disease was seen with a dehiscence of his left foot status post transmetatarsal amputation (TMA). He had an excisional debridement and underwent a DVA, with a biodegradable bilayer synthetic matrix (BBSM) placed over the excisional debridement where the dehiscence was. The sealing layer was removed after 3 weeks, revealing healthy granulation tissue that underwent minor debridement.

**Results:** The patient continued wound care as an outpatient. Day 45 shows the wound with healthy pink vascularized tissue. On day 60, the patient shows the wound is mostly closed, with some exposed pink tissue and a small area of open wound. A visit on post op day 90 revealed a fully healed wound with smooth contour and minor scarring.

**Conclusions:** This case shows that BBSM may be an appropriate wound closure method for surgical wounds on previously ischemic limbs that have undergone DVA, accommodating the impaired healing rate associated with impaired vascularization. Maturation of DVAs can take several months, with compromised wound healing during that time. BBSM's degradation profile allows it to stay in place during this timeline.

53

### 53.1 Efficacy of Intravenous versus Oral Discharge Antibiotics in Reducing Osteomyelitis Recurrence After Lower Extremity Free Tissue Transfer

#### 53.1.1 Rachel Rohrich; Christopher Ply; Hannah Soltani, BA; Esther Kang; Karen Kim Evans, MD

**Background:** Microsurgical free tissue transfer (FTT) effectively heals osteomyelitis by providing healthy vascularized tissue, but recurrence remains common in nontraumatic chronic lower extremity wounds. Recent studies suggest oral antibiotics may match IV therapy, though this hasn't been confirmed in this population. This study compares oral versus IV antibiotics post‐discharge in patients receiving LE FTT for osteomyelitis.

**Methods:** A retrospective review of all LE FTT performed by a single surgeon from February 2011 to July 2024. Patients with a positive bone culture (osteomyelitis) on the date of the FTT reconstruction were included. Patients were stratified by the route of antibiotics they were discharged with (Oral vs. IV). Primary outcome was osteomyelitis recurrence.

**Results:** A total of 179 LE FTT were included. The median age was 59.9 years (IQR:16.0) The rate of osteomyelitis recurrence was 24 (22.9%), insignificantly greater in the IV antibiotics group (31.1% vs 16.9%, p=0.089). Regression analysis revealed that no variables were significantly associated with osteomyelitis recurrence.

**Conclusions:** In patients undergoing LE FTT for the treatment of chronic wounds with active osteomyelitis, IV antibiotics upon discharge offer protective benefit compared to oral antibiotics in reducing osteomyelitis recurrence, even when controlling for baseline comorbidities.

54

### 54.1 Masked by Minimal Pain: Trauma‐Associated Group A Streptococcal Necrotizing Fasciitis in a Low‐Risk Patient Successfully Managed Through Aggressive Limb Salvage and Staged Reconstruction

#### 54.1.1 Bianca Boafo, DPM; Alex Fiola, DPM; Samantha Trepal, DPM; Russell Caprioli, DPM

**Background:** Necrotizing fasciitis is a rapidly progressive soft tissue infection with high risk of amputation and death. Atypical presentations make early diagnosis difficult. Up to 25 percent of patients lack traditional risk factors. Group A Streptococcus can cause low pain infections due to toxin related nerve injury and microvascular thrombosis. Trauma associated infection without open wounds or major medical co‐morbidities is a critical diagnostic pitfall and requires early surgical evaluation.

**Methods:** A 64 year old male presented after minor foot trauma with rapidly progressive erythema, edema, but minimal pain. He had no open wounds or major co‐morbidities. Labs showed leukocytosis with LRINEC score of 10. Imaging showed no gas, masking severity. Emergent surgery revealed extensive fascial necrosis, dishwater fluid, and loss of normal fascial planes with high amputation risk. Cultures grew Group A Streptococcus. Management required serial debridements, NPWT, and staged reconstruction.

**Results:** Despite extensive soft tissue necrosis and high amputation risk, staged reconstruction achieved progressive granulation, wound contraction, and near complete epithelialization without osteomyelitis. The patient avoided major amputation and achieved durable limb preservation with restoration of function, including return to weight bearing and full ambulation, allowing independence in activities of daily living at follow up.

**Conclusions:** This case highlights an uncommon presentation of trauma associated GAS necrotizing fasciitis with minimal pain, no open wounds, and misleading imaging in a low risk patient. Aggressive early surgical exploration combined with staged reconstruction can achieve limb salvage even in extensive necrotizing infection. Limb threatening soft tissue infections should be managed using structured limb salvage principles regardless of underlying cause or traditional risk factors.

55

### 55.1 Optimizing Uncontrolled Versus Controlled Diabetes Mellitus in the Perioperative Setting for Non‐ Traumatic Lower Extremity Free Tissue Transfer

#### 55.1.1 Finn Tobias, BS; Karen Li, MD; Hannah Soltani, BA; Rachel Rohrich, BS; Ryan Lin, MD; Christopher M. Ply, BS; Christopher E. Attinger, MD; Richard C. Youn, MD; Karen Kim Evans, MD

**Background:** Lower extremity (LE) limb salvage with free tissue transfer (FTT) has shown high success rates in a highly comorbid population as surgical techniques and experience improve. As patient selection expands to include greater comorbidity, preoperative medical optimization is increasingly critical. This study evaluates short‐ and long‐term outcomes of LE FTT in controlled versus uncontrolled diabetic patients at our institution.

**Methods:** Lower extremity FTTs performed by a single surgeon (2011–2025) were reviewed. Demographics, comorbidities, preoperative management, intraoperative details, immediate outcomes, short‐term complications, and long‐term outcomes were compared between controlled DM (HbA1c <7) and uncontrolled DM (HbA1c >7). Medial arterial calcification (MAC) was stratified as absent (0–1 sites), moderate (2–3), or severe (4–5).

**Results:** No differences in age, BMI, sex, or smoking were seen between uncontrolled (uDM) and controlled DM (cDM). uDM had higher PVD (40.3% vs 27.5%, p=0.002), pre‐FTT vascular intervention (26.4% vs 10.6%, p<0.001), and moderate/severe MAC (p=0.001). Immediate outcomes and flap success were similar (97.3% vs 96.5%, p=0.673). Hematoma (20.8% vs 12.3%, p=0.039) and long‐term amputation (21.5% vs 8.2%, p=0.001) were higher in uDM.

**Conclusions:** FTT remains an effective limb salvage option for patients with diabetes and vascular disease, with high flap success rates. However, patients with uncontrolled diabetes should be considered high risk and require closer perioperative and postoperative management to optimize long‐term outcomes. These findings emphasize the importance of multidisciplinary medical optimization throughout all stages of FTT care.

56

### 56.1 Dynamic Offloading Device: A Patient‐Centered Solution to Reduce Skin Breakdown

#### 56.1.1 Cameron Darnell, BA; Christopher Ply, BS; Hannah Soltani, BA; Paul Chao, PhD; John Steinberg, DPM; Jayson Atves, DPM; Richard Youn, MD; Christopher Attinger, MD; Karen Kim Evans, MD

**Background:** Diabetic foot ulcers remain a leading cause of lower extremity amputation, driven by neuropathy and focal plantar pressure that patients cannot perceive. Although offloading footwear is the cornerstone of prevention, clinicians lack objective methods to verify pressure redistribution. Improper offloading leads to recurrent skin breakdown and re‐ulceration, highlighting the need for real‐time, patient‐centered monitoring.

**Methods:** A patient‐developed, low‐cost dynamic offloading device was implemented to sense and quantify pressure during ambulation. Using force‐sensing resistors from Tactilus, the wearable sensor transmits real‐time pressure data via Bluetooth to a smartphone application, displaying pressure magnitude, gait cycles, and precise loading patterns. The system enables clinicians to guide orthotic adjustments and allows patients to modify gait in response to high‐pressure alerts.

**Results:** Two patients with neuropathy and prior amputations, one with a partial foot amputation and one with a transmetatarsal amputation, experienced recurrent plantar skin breakdown due to shifted load distribution. After implementing the pressure‐sensing device to guide orthotic modifications and continuous gait adjustments, both patients achieved effective offloading and have had no recurrence of ulceration during follow‐up.

**Conclusions:** Dynamic pressure monitoring provides an objective, patient‐centered method to verify offloading effectiveness and prevent recurrent skin breakdown in insensate patients. This low‐cost technology enables data‐driven orthotic optimization and real‐time behavioral feedback. Broader implementation may reduce ulcer recurrence, prevent limb loss, and offer an accessible solution for diabetic limb preservation and reconstructed lower extremities through real‐time pressure redistribution.

57

### 57.1 Outcomes of Flap Reconstruction for Diabetic Foot Ulcers: Our 14 Year Experience

#### 57.1.1 Hannah Soltani, BA; Priya Meesa, BA; Chrisopher Ply, BS; Leila I. Jones, BS; Christopher E. Attinger, MD; Richard C. Youn, MD; Karen K. Evans, MD

**Background:** Diabetic foot ulcers (DFUs) are a leading cause of lower extremity amputation and pose significant reconstructive challenges. We have demonstrated that free tissue transfer (FTT) offers durable soft tissue coverage and limb salvage in complex lower extremity wounds but have not characterized its use solely in DFUs. This study evaluates outcomes and complications following FTT for DFUs to better characterize its role in diabetic limb salvage.

**Methods:** We performed a retrospective review of patients undergoing flap reconstruction for DFUs at a tertiary care center between July 2011‐December 2025. Demographics, comorbidities, vascular status, wound characteristics, flap type, and perioperative variables were collected. Primary outcomes included flap success and limb salvage. Secondary outcomes included complications, reoperation, healing time, and mortality.

**Results:** A total of 219 patients were identified with DFUs undergoing FTT. Average age 58.1±10.7 years, HgA1c was 8.1±2.3%, and 51 (23.3%) of patients had chronic kidney disease. The majority of wounds were located at the hindfoot (30.6%) followed by the forefoot (30.0%). Immediate flap success was 95.9%, and 6.4% of flaps required takeback within 7 days, of which 93% were salvaged. Limb salvage was achieved in 81.3%. Functional ambulation was restored in 74% at an average final follow‐up of 26 months.

**Conclusions:** FTT provides reliable soft tissue coverage and high limb salvage rates in patients with DFUs. Despite significant comorbidity burden, successful outcomes can be achieved with multidisciplinary care and careful patient selection. Identification of risk factors for failure may improve surgical planning and optimize limb preservation strategies.

58

### 58.1 Following Rehabilitation Recommendations May Improve Outcomes in Highly Comorbid Patients Undergoing Lower Extremity Free Flap

#### 58.1.1 Parker Buck, MS; Christopher Ply; Hannah Soltani, BA; Nori Pham; Karen Kim Evans, MD

**Background:** After a lengthy hospitalization for free tissue transfer (FTT), many patients fail to receive the recommended level of rehabilitation. This study aims to identify the barriers and consequences of receiving an alternate level of rehabilitation than recommended.

**Methods:** A retrospective review was performed from 2015‐2025 of patients undergoing FTT for atraumatic lower extremity wounds at a tertiary wound center. Physical therapy (PT) and/or occupational therapy (OT) rehabilitation recommendations were compared to the level of rehabilitation actually received. Patients were stratified into rehabilitation match and mismatch groups. Baseline characteristics, comorbidities, and outcomes were compared.

**Results:** Of 350 patients receiving a lower extremity free flap, 73.4% had a rehabilitation match and 26.6% had a mismatch. Baseline comorbidities and insurance type did not differ. Mismatch reasons included patient refusal (31.3%), insurance denial (18.8%), lack of home PT (14.3%), and facility rejection (10.7%). Mismatch patients were more likely to be recommended acute rehabilitation (71.0% vs 51.6%) than home PT (19.4% vs 40.5%, p=0.015). Time to ambulation was increased but not significantly.

**Conclusions:** Nearly one‐third of FTT patients experience rehabilitation mismatch, primarily driven by nonclinical barriers. Although overall ambulation rates were similar, delayed mobilization trends in mismatch patients suggest that inadequate access to recommended rehabilitation may slow functional recovery. Addressing insurance authorization, facility acceptance, and discharge coordination represents a modifiable target to improve postoperative recovery timelines following limb salvage reconstruction.

59

### 59.1 Use of Muscle Versus Fasciocutaneous Free Flaps in Limb Salvage for Patients with Venous Reflux

#### 59.1.1 Samuel Huffman, MD; Karen Li, MD; Hannah Soltani, BA; Rachel Rohrich, BS; Christopher E. Attinger, MD; Richard C. Youn, MD; Karen K. Evans, MD

**Background:** The hemodynamics of venous reflux (VR) are not well studied in current lower extremity (LE) free tissue transfer (FTT) literature. In extremities with high outflow venous pressure or VR, there have been no studies to suggest a preferred free flap choice. Our study aims to compare the use of fasciocutaneous (FCFF) versus muscle free flaps (MFF) in patients with VR and evaluate the postoperative outcomes for those with venous disease.

**Methods:** A retrospective review of 114 patients with presence of VR on preoperative LE venous duplex ultrasound imaging were included. 69 (60.5%) patients underwent FCFF and 45 (39.5%) patients underwent MFF. Demographics, comorbidities, preoperative LE venous findings, and outcomes were collected. A sub‐analysis evaluated the outcomes of patients with arterial, venous, or both types of vascular disease.

**Results:** Patients that underwent MFF had a higher prevalence of diabetes (77.8% vs 53.6%, <0.001), chronic kidney disease (33.3% vs. 11.6%, p=0.005), and end stage renal disease (15.6% vs. 2.9%, p=0.028). Between MFF and FCFF cohorts, there were no significant differences in vessel run‐off (VRO) on the preoperative angiogram or presence of VR on preoperative venous testing. FCFF had higher rates of takeback (4.35% vs. 0.0%, p=0.156) and partial necrosis (5.8% vs. 0.0%, p=0.152).

**Conclusions:** For an increasingly comorbid patient population, characterizing arterial inflow and understanding venous pathology before free tissue transfer is important to achieve favorable outcomes. There may be a benefit in using MFF over FCFF for patients with venous disease, which theoretically could be due to higher flow mechanics in muscle to overcome higher backpressures for refluxed vessels.

60

### 60.1 The Use of the Omental Flap for Debilitating Chronic Lower Extremity Wounds

#### 60.1.1 Winnie Li, BS; Hannah Soltani, BA; Rachel N Rohrich, BS; Christopher Ply, BS; Jessica R. Ong, MD PhD; Karen Li, MD; Christopher E. Attinger, MD; Richard C. Youn, MD; Karen K. Evans, MD

**Background:** The omental flap's traditional use for sternal wound infection and pelvic reconstruction is well‐defined. However, its use in lower extremity (LE) reconstruction, where it may offer distinct advantages for complex wounds due to its robust vascular, immunologic, and lymphangiogenic properties, is less characterized. This study describes the use of omental flap for the coverage of atraumatic LE chronic wounds in a vasculopathic, highly comorbid population.

**Methods:** We performed a single‐institution retrospective review of adult patients undergoing LE free tissue transfer (FTT) with omental flaps from 2011‐2025. For such patients, the omental flap was chosen largely due to the size of the wound. Demographics, comorbidities, preoperative angiographic findings, intraoperative details, and postoperative outcomes were collected. Primary outcomes included flap success and limb salvage.

**Results:** Four patients (mean age 65.8 ± 10.6 years; 50% male) underwent omental FTT for chronic LE wounds (median wound area 180 cm^2^). All had significant comorbidities including lymphedema (75%), peripheral artery disease (75%), and chronic osteomyelitis (75%). Flap success was achieved in all cases, with one instance of takeback (salvaged) and partial flap necrosis (25%). At an average follow‐up of 8.5 ± 4.2 months, all patients achieved full ambulation and functional limb salvage.

**Conclusions:** Omental flaps offer a reliable option for large, chronic LE wounds, combining durable coverage with immunologic and lymphatic benefits, and may help achieve limb salvage in high‐risk patients while maintaining acceptable donor site morbidity.

61

### 61.1 Free Tissue Transfer is Associated with Long‐Term Survival Advantage over Below Knee Amputation or Local Flap Reconstruction in the Setting of High Medial Arterial Calcification Burden

#### 61.1.1 Winnie Li, BS; Hannah Soltani, BA; Rachel N Rohrich, BS; Christopher Ply, BS; Ryan Lin, MD; Christopher E. Attinger, MD; Richard C. Youn, MD; Karen K. Evans, MD

**Background:** Medial arterial calcification (MAC) has been linked to poor limb salvage and technical challenges during free tissue transfer (FTT). Local flaps (LF) may offer comparable salvage in high‐risk patients, while some ultimately require below‐knee amputation (BKA). The effect of MAC burden on long‐term mortality across these treatments remains unclear. This study evaluates 5‐year mortality following BKA, LF, and FTT in patients with MAC.

**Methods:** We performed a retrospective cohort study of patients who underwent either FTT, LF, or primary BKA at a tertiary care wound center between January 2012 and June 2020. Two independent reviewers evaluated pedal radiographs and assigned MAC scores using the Ferrassi grading scale, a validated tool for assessing medial arterial calcification severity. Mortality rates in patients who had high MAC (4‐5) were then compared based on index surgery (FTT, LF, or BKA).

**Results:** Of 775 patients who underwent FTT, LF, or BKA, 133 had high MAC (FTT n = 24 [18.1%]; LF n= 42 [31.6%]; BKA n = 67 [50.4%]). ESRD rates were significantly lower in FTT (29.2%) compared to LF (59.5%, p=0.018) and BKA (70.2%, p=0.001). Proximal amputation rates were similar between FTT (33.3%) and LF (30.1%, p=0.842). FTT had significantly lower 5‐year mortality (12.5%) compared to LF (40.5%, p=0.015) and BKA (44.8%, p=0.004). High MAC and ESRD were independent predictors of 5‐year mortality.

**Conclusions:** Among patients with high MAC burden, FTT may confer a survival benefit compared to other reconstructive strategies. Severe MAC and ESRD independently predicted mortality, underscoring the importance of risk stratification when selecting limb salvage strategies.

62

### 62.1 Iterative “Vasculo‐Plastic” Protocol Refinement Enables Limb Salvage in a Growing Vasculopat

#### 62.1.1 Rachel Rohrich BS, Hannah Soltani BA, Karen Li, MD, Leila I. Jones, BS; Meghan Currin, BS; Christopher E. Attinger, MD; Richard C. Youn, MD; Cameron Akbari, MD; Karen K. Evans, MD

**Background:** Contemporary limb salvage increasingly involves patients with severe peripheral vascular disease (PVD), diabetes, and medial arterial calcification (MAC), yet few studies describe how microsurgical programs have adapted to this evolving complexity. Over 14 years, our institution has performed 400 consecutive lower extremity (LE) free tissue transfers (FTT). This study quantifies the longitudinal impact of protocol evolution on patient selection, microsurgical technique, and clinical outcomes.

**Methods:** A prospectively maintained database (2011‐2025) was stratified into four sequential 100‐case cohorts: (1) initial practice era (2011‐2017), (2) early established era (2017‐2020), (3) late established era (2020‐2023), and (4) current practice era (2023‐2025). Era‐specific milestones included risk‐stratified anticoagulation, intraoperative Doppler and tissue oximetry monitoring, saphenous vein interposition grafting, co‐surgeon approach, and preoperative MAC screening with tibial radiography.

**Results:** Patient complexity increased significantly over time, with high rates of diabetes (56%), PVD (58%) and MAC (16%) in the current practice era. Median operative duration decreased (p<0.001). Immediate flap success increased from 93.0% to 99.0% (p=0.050). Median time to independent ambulation decreased significantly from the initial to current practice era (p<0.001). Limb salvage rates remained =88.0% across all eras (p=0.069) at a median follow‐up of 16.9 (IQR: 26.4) months in the total cohort.

**Conclusions:** Over 14 years, our program has evolved through adjustments in perioperative protocols and microsurgical technique, enabling safe limb salvage in an increasingly complex patient population. In the most recent era, marked by the highest burden of comorbidities, flap success and limb salvage were not compromised. The success and progression of this program illustrates that sustained improvement requires iterative change, and as clinical demands intensify, evolution of practice must continue.

63

### 63.1 Characteristics of a Hospital System's Limb Loss and Preservation Data

#### 63.1.1 Robert Skerker, MD

**Background:** The Limb Loss and Preservation Registry (LLPR) is a national data quality repository built to collect data to improve care for patients affected by limb loss. The goal is to understand the level of care patients are receiving across the continuum. Atlantic Health (AH) is a large NJ health care system that has a commitment to build healthier communities and improve lives for patients. AH has partnered with the LLPR to collect data that will allow the organization to study the care given.

**Methods:** Quantify demographic and medical details amongst cohorts of patients, and analyze the care given, to gain baseline information that can be used to study outcomes with the objective of improving care across the continuum to prevent limb loss when possible; and if not, then improve post limb loss outcomes. This abstract is AH's first attempt at presenting preliminary data recently acquired, as we delve deeper into the data and improve our ability to analyze it with the help of the LLPR.

**Results:** 1. Patient Demographics

• Total Patients: [Insert]

• Race/Ethnicity Breakdown: [Insert]

• Insurance Status: [Insert]

2. Clinical Outcomes

• Limb Salvage Rate: [Insert %]

• Major Amputation Rate (Transtibial and proximal): [Insert %]

• Minor Amputation Rate (Ankle and distal): [Insert %]

3. Comorbidity Profile

• Diabetes: [Insert %]

• Peripheral Artery Disease (PAD): [Insert %]

• Chronic Kidney Disease (CKD): [Insert %]

**Conclusions:** The Limb Loss and Preservation Registry (LLPR) is the first national data quality repository built to collect real‐world data to improve care for patients affected by limb preservation and/or limb loss. Data extraction and analysis will aide acquisition of health quality metrics that will be used to improve the quality of care for this population cohort and also help identify marginalized groups for whom strategies could be developed to improve outcomes.

## Conflicts of Interest

The authors declare no conflicts of interest.

## Data Availability

The data that support the findings of this study are available on request from the corresponding author. The data are not publicly available due to privacy or ethical restrictions.

